# Normative mice retinal thickness: 16-month longitudinal characterization of wild-type mice and changes in a model of Alzheimer's disease

**DOI:** 10.3389/fnagi.2023.1161847

**Published:** 2023-04-06

**Authors:** Ana Batista, Pedro Guimarães, João Martins, Paula I. Moreira, António Francisco Ambrósio, Miguel Castelo-Branco, Pedro Serranho, Rui Bernardes

**Affiliations:** ^1^Coimbra Institute for Biomedical Imaging and Translational Research (CIBIT), Institute for Nuclear Sciences Applied to Health (ICNAS), University of Coimbra, Coimbra, Portugal; ^2^Coimbra Institute for Clinical and Biomedical Research (iCBR), Faculty of Medicine (FMUC), University of Coimbra, Coimbra, Portugal; ^3^Center for Innovative Biomedicine and Biotechnology (CIBB), University of Coimbra, Coimbra, Portugal; ^4^Clinical Academic Center of Coimbra (CACC), Faculty of Medicine (FMUC), University of Coimbra, Coimbra, Portugal; ^5^Laboratory of Physiology, Faculty of Medicine (FMUC), University of Coimbra, Coimbra, Portugal; ^6^Center for Neuroscience and Cell Biology (CNC), University of Coimbra, Coimbra, Portugal; ^7^Mathematics Section, Department of Sciences and Technology, Universidade Aberta, Lisbon, Portugal

**Keywords:** optical coherence tomography, retinal thickness, normative data, Alzheimer's disease, 3 × Tg-AD animal model

## Abstract

Animal models of disease are paramount to understand retinal development, the pathophysiology of eye diseases, and to study neurodegeneration using optical coherence tomography (OCT) data. In this study, we present a comprehensive normative database of retinal thickness in C57BL6/129S mice using spectral-domain OCT data. The database covers a longitudinal period of 16 months, from 1 to 16 months of age, and provides valuable insights into retinal development and changes over time. Our findings reveal that total retinal thickness decreases with age, while the thickness of individual retinal layers and layer aggregates changes in different ways. For example, the outer plexiform layer (OPL), photoreceptor inner segments (ILS), and retinal pigment epithelium (RPE) thickened over time, whereas other retinal layers and layer aggregates became thinner. Additionally, we compare the retinal thickness of wild-type (WT) mice with an animal model of Alzheimer's disease (3 × Tg-AD) and show that the transgenic mice exhibit a decrease in total retinal thickness compared to age-matched WT mice, with statistically significant differences observed at all evaluated ages. This normative database of retinal thickness in mice will serve as a reference for future studies on retinal changes in neurodegenerative and eye diseases and will further our understanding of the pathophysiology of these conditions.

## 1. Introduction

Optical coherence tomography (OCT), first demonstrated in 1991, is a non-contact imaging modality based on interferometry. It was first used to image the human retina *in vivo* in 1993 (Swanson et al., 1993). Since then, OCT has transformed medical retinal imaging. The fact that OCT is a high-resolution, non-invasive modality with fast scan times are just a few of the features that have facilitated the introduction and expansion of OCT into clinical practice.

Currently, OCT devices are available in most clinics and are widely used to evaluate the retina in healthy and diseased conditions. It is used to aid in the diagnosis of ophthalmological complications, such as diabetic retinopathy, macular edema, and age-related macular degeneration. Moreover, OCT has been at the forefront of retinal biomarkers of neurodegeneration research (Doustar et al., 2017; Vujosevic et al., 2023). Because of the clear optics of the eye, together with the fact that the retina is part of the central nervous system, OCT offers a direct, quick, and inexpensive method to image neurodegeneration. Magnetic resonance imaging (MRI) and positron emission tomography (PET) remain the gold standard in diagnosing neurodegeneration. However, these modalities are expensive, cumbersome, and difficult to access.

OCT-based *in vivo* human research to find novel biomarkers of neurodegeneration has a major limitation: patients are diagnosed in the late stages of the disease, and the time of onset is difficult to ascertain. Therefore, mouse models of disease are often chosen as surrogates. Mouse models have been important in multiple areas of research and have improved our understanding of disease pathophysiology. Based on OCT imaging, retinal changes induced by neurodegenerative diseases have been found, including changes in retinal layer thickness (Cunha et al., 2016; Song et al., 2020; Salobrar-García et al., 2021). Recently, we have also shown that retinal texture biomarkers can help discriminate between age-matched healthy controls and animal models of Alzheimer's disease (AD) in the early stages (Nunes et al., 2019; Ferreira et al., 2020, 2022; Guimarães et al., 2022).

Although mouse models of disease play a key role in clinical research, there is still a lack of longitudinal normative data on retinal thickness in mouse models. Normative data have been focused on thickness alterations in the first months of mouse development, leaving out long-term changes. Ferguson et al. (2013) reported on normative retinal thickness data from one of the most commonly used mouse strains of wild-type (WT) mice (C57BL/6). Thickness values of different retinal layers and total retinal thickness were reported in four retinal regions (nasal, temporal, superior, and inferior). The 30 animals included in this study were between 3 and 5 months of age (Ferguson et al., 2013). Total retinal and layer thicknesses are age dependent. Thus, averaging animals with a non-discriminatory wide range of ages leads to incorrect generalizations of normative data. Furthermore, the establishment of a single normative time point hinders longitudinal efforts. Recently, our group reported on normative data of WT mice (strain C57BL6/129S) and of the triple transgenic mouse model with three mutated human genes associated with familial AD (3 × Tg-AD): Swedish amyloid precursor protein (APPswe); presenilin 1 (PSEN1); microtubule-associated protein tau (MAPT). Longitudinal retinal thicknesses, differentiated by age and eye, were presented for both groups of animals up to 4 months of age (Ferreira et al., 2021). Although some neuronal changes have been reported in the early stages of the disease, such as amyloid beta immunoreactivity at 2 months, the accumulation of extracellular senile plaques does not become apparent until 6 months of age, while neurofibrillary tangles are not detectable until 3 × Tg-AD animals are 12 months old (Sterniczuk et al., 2010; Javonillo et al., 2022). Thus, characterization of the retina over a longer period of time is paramount. Here, we characterize the total retinal thickness and the thickness of individual layers for WT and 3 × Tg-AD animals aged up to 16 months. The same animals were monitored longitudinally over time. The thickness of the retina and retinal layers are discussed as a function of age and the relative distance to the optic disc. Moreover, 3 × Tg-AD induced changes in retinal thickness are reported.

## 2. Material and methods

### 2.1. Animals

A total of 114 male mice were used in this study: 57 WT (C57BL6/129S) and 57 genetically modified (3 × Tg-AD). The former is a strain that contains a 129-derived genetic interval encompassing the *Gbp* gene cluster on a C57BL/6 genetic background, and the latter a mouse model of familiar AD with three mutated human genes. For each animal, both eyes were imaged at 1, 2, 3, 4, 8, 12, and 16 months of age. Some retinal thickness data were excluded due to subpar OCT data or segmentation errors. Detailed information on the number of OCT data volumes of animals per experimental group at each time point and differentiated by eye is shown in Table 1.

**Table 1:** Total number of OCT data volumes included in this study for wild-type (WT) mice and the triple transgenic Alzheimer's disease (3 × Tg-AD) mice model, differentiated by age and eye.

**Age (months)**		**1**	**2**	**3**	**4**	**8**	**12**	**16**
WT	OS	56	51	51	53	49	46	32
	OD	53	51	49	53	49	44	27
3 × Tg-AD	OS	54	53	53	51	47	43	35
	OD	55	54	54	51	46	43	43

OD and OS denote oculus dexter and oculus sinister, respectively.

Between data acquisitions, animals were maintained in an animal house facility at the Coimbra Institute for Clinical and Biomedical Research (iCBR), Faculty of Medicine, University of Coimbra. Animals were kept in rooms with controlled temperature, reverse light cycles (12-h light/dark), and free access to water and food. Body weight of the animals was monitored throughout the experimental period. Mean animal weights (and standard deviations) for both groups at all ages are shown in Table 2. A significant increase in animal body weight with age, evaluated by repeated measures ANOVA, was observed for both groups (*p*-values < 0.001 for both groups). No statistical differences were observed between the weights of WT and 3 × Tg-AD mice up to 4 months of age. For mice with 8 months of age or older, WT mice had significantly higher body weights than 3 × Tg-AD mice. Statistical differences were assessed by independent samples *t*-test.

**Table 2:** Longitudinal average weight distribution of wild-type (WT) and the triple transgenic Alzheimer's disease (3 × Tg-AD) mice.

**Age (months)**	**1**	**2**	**3**	**4**	**8**	**12**	**16**
WT	14.82 (2.62)	22.63 (1.68)	25.25 (1.91)	26.95 (2.09)	32.69^*^ (3.53)	35.04^*^ (3.46)	36.98^*^ (4.09)
3 × Tg-AD	14.90 (2.64)	22.24 (2.32)	25.60 (2.24)	27.49 (2.04)	30.41 (2.84)	31.66 (3.48)	32.91 (3.69)

Weight data are expressed in grams as mean (standard deviation). Statistical differences between the two groups, evaluated by the independent samples t-test are also indicated (^*^p-value < 0.001).

For OCT data acquisition, animals were anesthetized with a mixture of 80 mg/kg of ketamine (Nimatek; Dechra) and 5 mg/kg xylazine (Sedaxylan; Dechra). A solution of 0.5% tropicamide (Tropicil; Edol) and 2.5% phenylephrine (Davinefrina; Dávi) was used for pupil dilation, and oxibuprocaine (Anestocil; Edol) was used for topical anesthesia. During OCT acquisition, the eyes were regularly lubricated with eye drops [1% carmellose (Celluvisc; Allergan)].

This study was approved by the Animal Welfare Committee of iCBR. All experiments were conducted in accordance with the Association for Research in Vision and Ophthalmology statement for animal use in research applications, and in agreement with the European Community Directive Guidelines for the care and use of non-human animals for scientific purposes (2010/63/EU), transposed into the Portuguese law in 2013 (DL113/2013).

### 2.2. OCT data acquisition

Retinal OCT data were acquired with a Micron IV OCT System (Phoenix Technology Group, Pleasanton, CA, USA). Each data volume consisted of 512 B-scans, each composed by 512 A-scans of 1024 discrete samples. B-scans were saved as non-compressed TIFF image files. The system uses a superluminescent diode emitting at a central wavelength of 830 nm with a bandwidth of 160 nm. The imaging depth of the system is 1.4 mm, with an axial resolution of 3 μm.

For consistency, all OCT data acquisitions were performed by the same operator. The optic disc was used as a landmark to select the imaged retinal region, which was horizontally aligned with and directly above it.

### 2.3. Automatic retinal segmentation

The retinal layer segmentation was determined using a deep learning approach. The neural network model used is detailed in Bernardes et al. (2022). In brief, it consists of a fully convolutional neural network (FCNN) following a U-type architecture (Ronneberger et al., 2015). The network consists of two main paths: an encoding and a decoding path, which combine global with local information to achieve a more accurate segmentation. In the encoding path, the feature maps' size was reduced at each level to compute increasingly global features. These features are then employed in the decoding path. The global features are expanded and used to classify each discrete sample in one of the retinal layers. Local features, obtained along the encoding and decoding paths via short-circuit connections (Ronneberger et al., 2015), are used to achieve finer-grain information and increase the accuracy of classification prediction.

**Figure 1:**
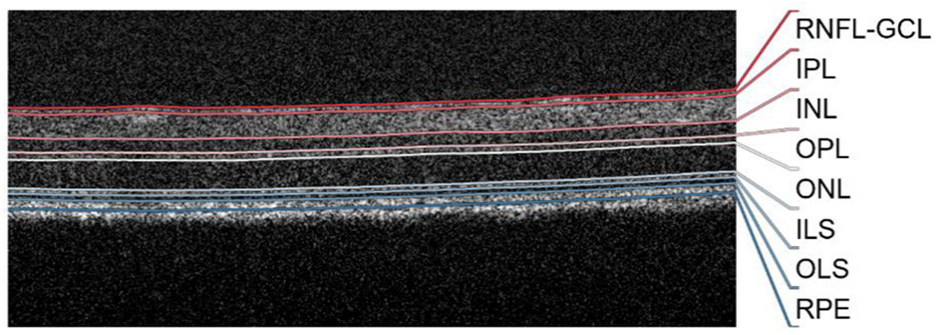
Representative B-scan with overlayed segmentation. RNFL-GCL, retinal nerve fiber layer and ganglion cell layer complex; IPL, inner plexiform layer; INL, inner nuclear layer; OPL, outer plexiform layer; ONL, outer nuclear layer; ILS, photoreceptor inner segments; OLS, photoreceptor outer segments; RPE, retinal pigment epithelium; TRT, total retina thickness.

A total of eight distinct structures (layers and layer aggregates) were considered in the classification (Figure 1): retinal nerve fiber layer and ganglion cell layer complex (RNFL-GCL), inner plexiform layer (IPL), inner nuclear layer (INL), outer plexiform layer (OPL), outer nuclear layer (ONL), photoreceptor inner segments (ILS), photoreceptor outer segments (OLS), and the retinal pigment epithelium (RPE). Volumetric segmentation is obtained by combining the 512 segmented B-scans.

#### 2.3.1. Retinal layer thickness

Retinal thickness maps were computed from the volumetric segmented OCT data. The thickness of each segmented structure and the total retinal thickness (TRT) were calculated as the distance between the respective segmented boundaries.

For each volume, nine regions-of-interest (ROIs) were considered to assess thickness variations within the imaged area. These consisted of 3 × 3 non-overlapping blocks of 170 × 170 points. The blocks, organized from temporal to nasal and superior to inferior directions, were labeled B1–B9, as described in Ferreira et al. (2021). The original data was cropped centrally into a 510 × 510 volume for ROI size consistency. Average thickness values were calculated as the average of the 510 × 510 and 170 × 170 thickness values for the entire imaged area and each ROI, respectively.

The segmentation quality of each thickness map value was evaluated prior to calculating the average thickness map based on three criteria: contrast between adjacent surfaces (data quality), segmentation (boundary) consistency, and distribution of the measured thicknesses. For each individual A-scan, failure to meet any criterion resulted in the exclusion of the A-scan from the analysis. Additionally, ROIs were completely excluded if more than 10% of all A-scans were discarded. The average exclusion percentages for B1 to B9 were: 9.4%, 5.7%, 6.0%, 8.2%, 4.9%, 6.1%, 8.6%, 5.7%, and 5.1%. The exact exclusion percentages for WT and 3 × Tg-AD discriminated per eye are detailed in Supplementary Table 1.

Data acquired from left and right eyes show nasal-temporal asymmetry. For a straightforward comparison between them, OCT scans from the left eyes were mirrored. Thus, left-hand-side/right-hand-side thickness maps correspond to the temporal/nasal region.

### 2.4. Statistical analysis

Statistical analysis was performed with Matlab R2020a (The MathWorks Inc., Natick, MA, USA). The normal distribution of the data was assessed using the Kolmogorov-Smirnov normality test, with a significance level of 10%. Over 93% of all thickness distributions were normal.

Statistical differences between the WT and 3 × Tg-AD groups were assessed using the independent samples *t*-test when normal distribution was confirmed. The alternative non-parametric Mann–Whitney *U*-test was used when the data did not follow a Gaussian distribution. Because multiple layers were compared at each time point, the Bonferroni correction was applied to correct for multiple comparisons.

For the analysis of statistical differences over time, one-way repeated measures ANOVA (RANOVA) or the non-parametric Friedman test were used depending on the normality of the data distribution. Only eyes that could be measured at all ages were included in this analysis (WT OS *N* = 27; WT OD *N* = 22; 3 × Tg-AD OS *N* = 33; 3 × Tg-AD OD *N* = 36). Two-way RANOVA was used to test the influence of each group on thickness values over time. Pairwise comparisons were evaluated and corrected for multiple comparisons using the Tukey–Kramer test. Significance levels of 5%, 1%, and 0.1% were considered.

## 3. Results

### 3.1. Total retinal thickness maps

**Figure 2:**
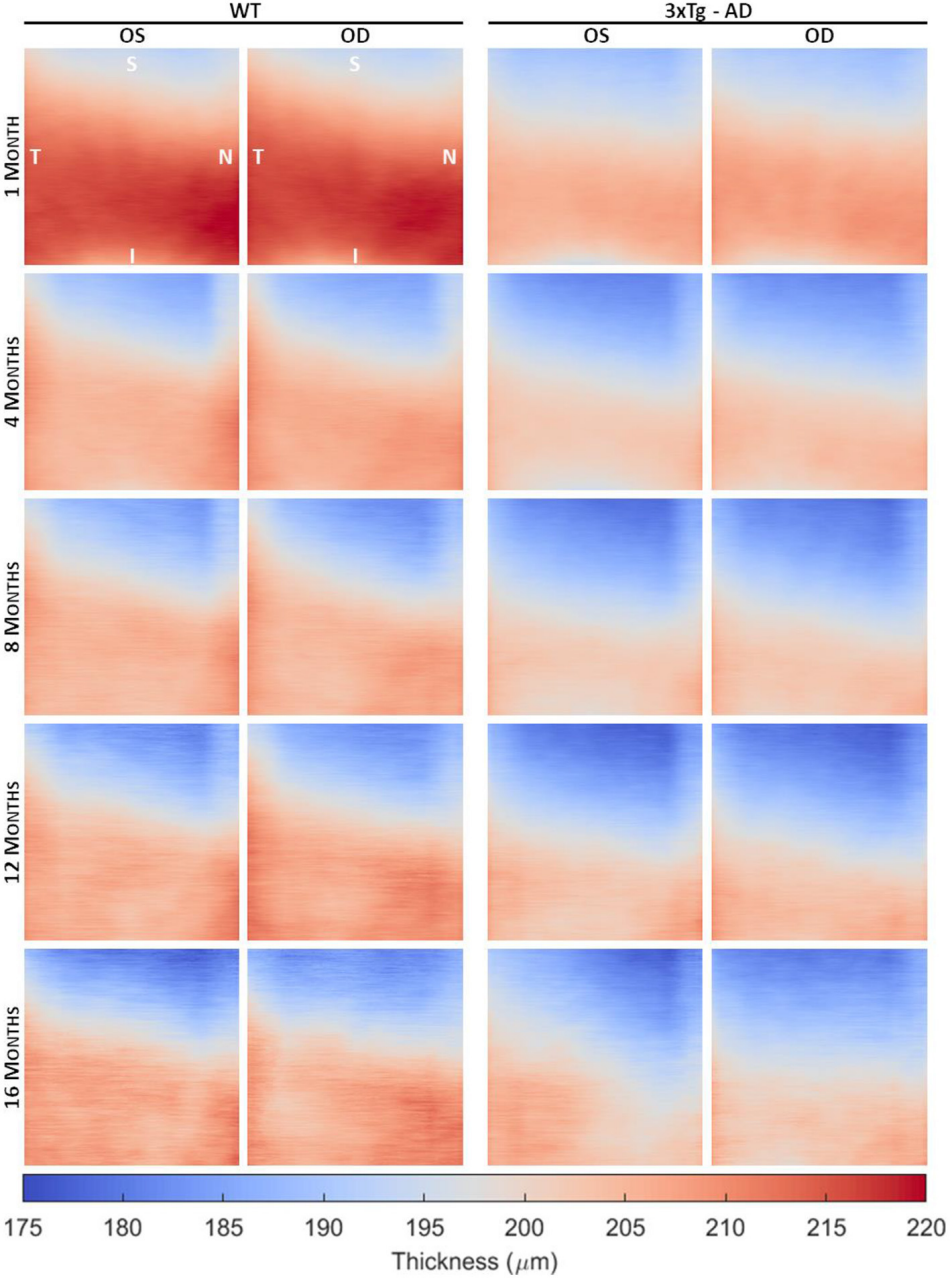
Average total retinal thickness maps of wild-type (WT) and the triple transgenic mouse model of Alzheimer's disease (3 × Tg-AD) retinas separated by left (OS) and right (OD) eyes of animals aged 1, 4, 8, 12, and 16 months. Color represents the retinal thickness in μm as indicated by the color bar. S, superior; I, inferior; T, temporal; N, nasal.

From the volumetric segmented OCT data of each subject, detailed thickness maps over the imaged area were computed for each retinal layer/layer aggregate. Figure 2 shows the average TRT maps, defined as the distance between the vitreous-RNFL and RPE-choroid interfaces, for WT and 3 × Tg-AD mice at 1, 4, 8, 12, and 16 months of age, differentiated by eye. The range of thickness values is similar for both eyes within the same group. Detailed analysis of the mean TRT values obtained for each eye at each time point showed no statistically significant differences in retinal thickness between the right and left eyes except for 3 × Tg-AD mice at 1 month of age (*p*-value = 0.02).

Detailed TRT maps indicate a decrease in overall retinal thickness with age in both groups. This decrease appears to be more pronounced between 1 and 2 months of age. Higher thickness values are observed in WT mice compared to 3 × Tg-AD mice for all ages. An increase in TRT can also be seen in both eyes with the proximity to the optic disc (superior to inferior). Additionally, an apparent temporal-nasal asymmetry was observed in both groups, with the temporal TRT appearing thicker.

### 3.2. Mean thickness

**Figure 3:**
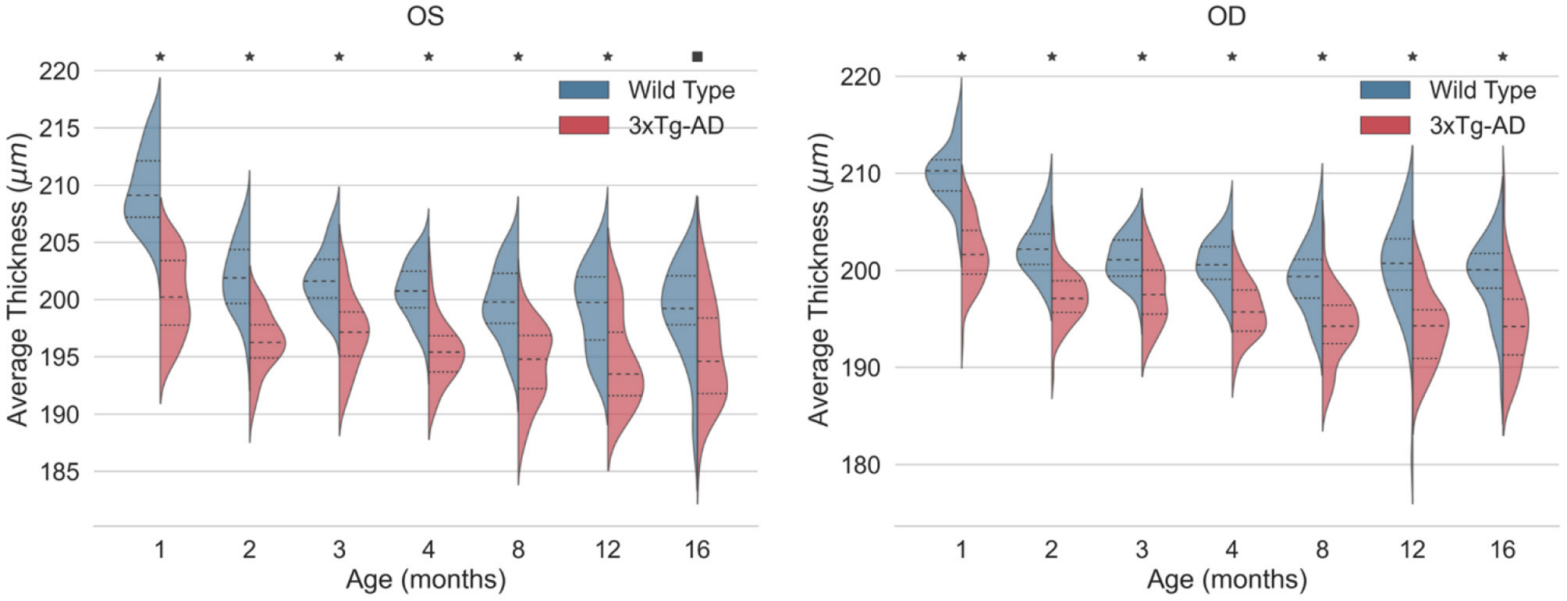
Kernel density estimates of the total retinal thickness (TRT) for the left eye (OS) and right eye (OD) of wild-type (blue) and the triple transgenic Alzheimer's disease (3xTg-AD) mice (red). Median (dashed) and first and third quartiles (dotted) are shown. *p*-values < 0.01 (■) and < 0.001 (*).

**Figure 4:**
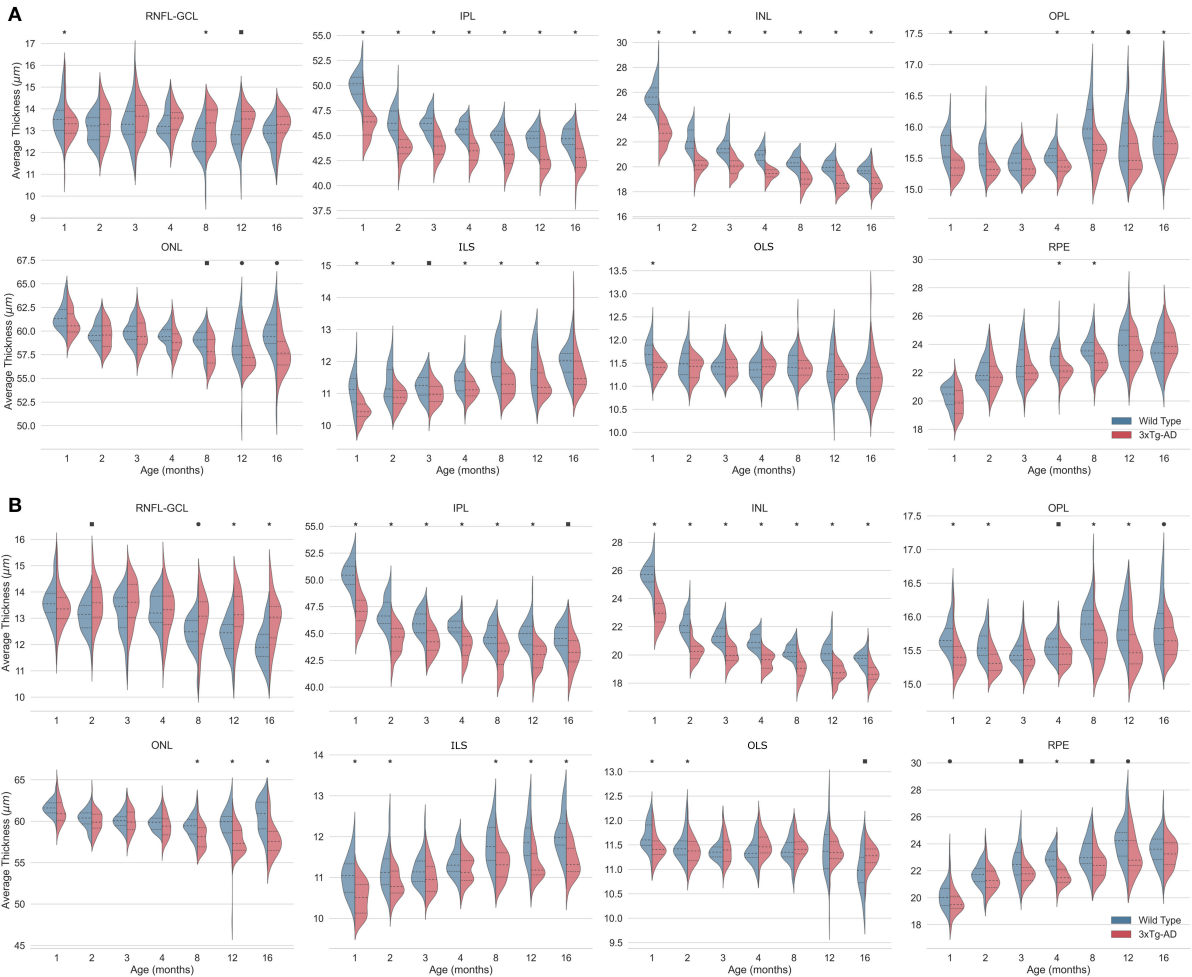
Kernel density estimates of retinal thickness per layer for the left **(A)** and right **(B)** eyes of wild-type (blue) and the triple transgenic Alzheimer's disease (3xTg-AD) mice (red). Median (dashed) and first and third quartiles (dotted) are shown. Bonferroni correction was applied per time point to correct for multiple comparisons. *p*-values < 0.05 (•), < 0.01 (■), and < 0.001 (*). RNFL-GCL, retinal nerve fiber layer and ganglion cell layer complex; IPL, inner plexiform layer; INL, inner nuclear layer; OPL, outer plexiform layer; ONL, outer nuclear layer; ILS, photoreceptor inner segments; OLS, photoreceptor outer segments; RPE, retinal pigment epithelium.

Detailed mean and standard deviation thickness values of the entire imaged area for WT and 3 × Tg-AD are shown in Table 3. Data from both eyes for each retinal layer/layer aggregate and the TRT were considered. Kernel density distributions of the TRT WT and 3 × Tg-AD mice for the left and right eyes are shown in Figure 3. Figure 4 shows the kernel density distributions of the retinal thickness for the eight retinal layers/layer aggregates considered for the left (Figure 4A) and right (Figure 4B) eyes. Supplementary Tables 2, 3 show detailed mean and standard deviation thickness values per mouse group, retinal layer, and age for the left and right eyes, respectively.

**Table 3:** Longitudinal thickness values, in μm, for both eyes of wild-type (WT) and the triple transgenic Alzheimer's disease (3xTg-AD) mice.

	**Age (months)**	**1**	**2**	**3**	**4**	**8**	**12**	**16**
WT	RNFL-GCL	13.59 (0.76)	13.10 (0.63)	13.27 (0.87)	13.32 (0.63)	12.51 (0.71)	12.62 (0.80)	12.34 (0.79)
	IPL	50.26 (1.33)	46.66 (1.43)	46.04 (1.15)	45.63 (0.85)	44.90 (1.09)	44.80 (1.18)	44.65 (1.14)
	INL	25.72 (0.99)	22.18 (0.86)	21.52 (0.76)	20.96 (0.57)	20.32 (0.56)	20.08 (0.76)	19.66 (0.61)
	OPL	15.71 (0.24)	15.58 (0.24)	15.46 (0.18)	15.56 (0.17)	15.96 (0.35)	15.84 (0.38)	15.89 (0.41)
	ONL	61.57 (1.11)	60.00 (1.16)	60.03 (1.12)	59.63 (0.97)	59.15 (1.25)	59.14 (2.33)	60.05 (2.21)
	ILS	11.06 (0.50)	11.25 (0.52)	11.21 (0.37)	11.38 (0.36)	11.90 (0.56)	11.88 (0.62)	12.04 (0.50)
	OLS	11.69 (0.30)	11.49 (0.30)	11.40 (0.23)	11.37 (0.25)	11.42 (0.30)	11.36 (0.46)	11.04 (0.40)
	RPE	20.26 (0.91)	21.91 (0.96)	22.62 (1.24)	22.97 (0.90)	23.41 (1.11)	24.11 (1.44)	23.51 (1.21)
	TRT	209.85 (3.08)	202.18 (2.89)	201.54 (2.46)	200.82 (2.48)	199.57 (3.33)	199.83 (4.39)	199.17 (3.96)
3 × Tg-AD	RNFL-GCL	13.37 (0.77)	13.45 (0.81)	13.57 (0.83)	13.36 (0.77)	13.10 (0.85)	13.31 (0.76)	13.08 (0.79)
	IPL	46.69 (1.52)	44.14 (1.28)	44.15 (1.23)	43.67 (1.22)	43.13 (1.34)	42.89 (1.31)	43.09 (1.56)
	INL	22.86 (0.97)	20.19 (0.68)	20.06 (0.68)	19.49 (0.54)	19.03 (0.72)	18.73 (0.67)	18.73 (0.75)
	OPL	15.39 (0.23)	15.34 (0.17)	15.38 (0.20)	15.41 (0.17)	15.59 (0.29)	15.55 (0.33)	15.74 (0.34)
	ONL	61.03 (1.40)	59.73 (1.32)	59.80 (1.45)	59.17 (1.42)	58.05 (1.52)	57.62 (1.53)	57.84 (2.00)
	ILS	10.48 (0.36)	10.87 (0.33)	11.00 (0.36)	11.16 (0.30)	11.31 (0.38)	11.31 (0.40)	11.57 (0.58)
	OLS	11.42 (0.23)	11.38 (0.25)	11.40 (0.25)	11.46 (0.24)	11.42 (0.24)	11.34 (0.24)	11.24 (0.39)
	RPE	19.80 (0.94)	21.58 (0.98)	21.96 (0.88)	21.87 (0.76)	22.60 (0.96)	23.43 (1.32)	23.68 (1.16)
	TRT	201.03 (3.47)	196.67 (2.62)	197.34 (3.21)	195.58 (2.68)	194.23 (3.36)	194.18 (3.81)	194.98 (4.51)

Data are expressed as mean (standard deviation).

RNFL-GCL, retinal nerve fiber layer and ganglion cell layer complex; IPL, inner plexiform layer; INL, inner nuclear layer; OPL, outer plexiform layer; ONL, outer nuclear layer; ILS, photoreceptor inner segments; OLS, photoreceptor outer segments; RPE, retinal pigment epithelium; TRT, total retina thickness.

Consistent statistically significant differences were found between WT and 3 × Tg-AD mice retinal layer thicknesses at all ages. Overall, the retina of 3 × Tg-AD mice is thinner than that of WT mice. Both eyes show significantly lower TRT values in 3 × Tg-AD mice than in WT mice at all time points (Figure 3).

Detailed analysis of individual layers shows that the RNFL-GCL complex of 3 × Tg-AD mice is thicker than that of WT mice. For the remaining retinal layers, the retinal layer thickness of WT mice is greater than that of 3 × Tg-AD mice. Consistently, statistically thinner IPL and INL layers were found in the retinas of 3 × Tg-AD mice at all ages in both eyes (Figure 4). The OPL was found to be statistically thinner in 3 × Tg-AD mice retinas than in WT mice retinas at all ages except at 3 months (Figure 4). Statistical differences between groups in the thickness of the RNFL-GCL complex and the ONL were observed primarily in mice aged 8 months or older.

Although a steady and continuous decrease in TRT was observed over time in both groups, the behavior of individual layers differs. A reduction of 4.9 and 3.1% was found in the TRT between mice aged 1 and 16 months for WT and 3 × Tg-AD, respectively. TRT thickness variations observed over the timespan covered result from a balance between thinning of the RNFL-GCL, IPL, INL, ONL, and OLS, combined with the thickening of the OPL, ILS, and RPE. In both groups, the layers with the greatest decrease in thickness were the INL (WT = 23.0 ± 2.9% and 3 × Tg-AD = 18.0 ± 4.6%) and IPL (WT = 11.3 ± 2.6% and 3 × Tg-AD = 7.8 ± 3.6%), whereas the highest increase was observed in the RPE (WT = 16.8 ± 8.5% and 3 × Tg-AD = 19.6 ± 7.6%) and ILS (WT = 9.1 ± 5.3% and 3 × Tg-AD = 10.1 ± 6.4%). The RNFL-GCL, OPL, ONL, and OLS have small to moderate thickness variations. Detailed variations of each layer thickness over the timespan covered are shown in Supplementary Table 4.

**Figure 5:**
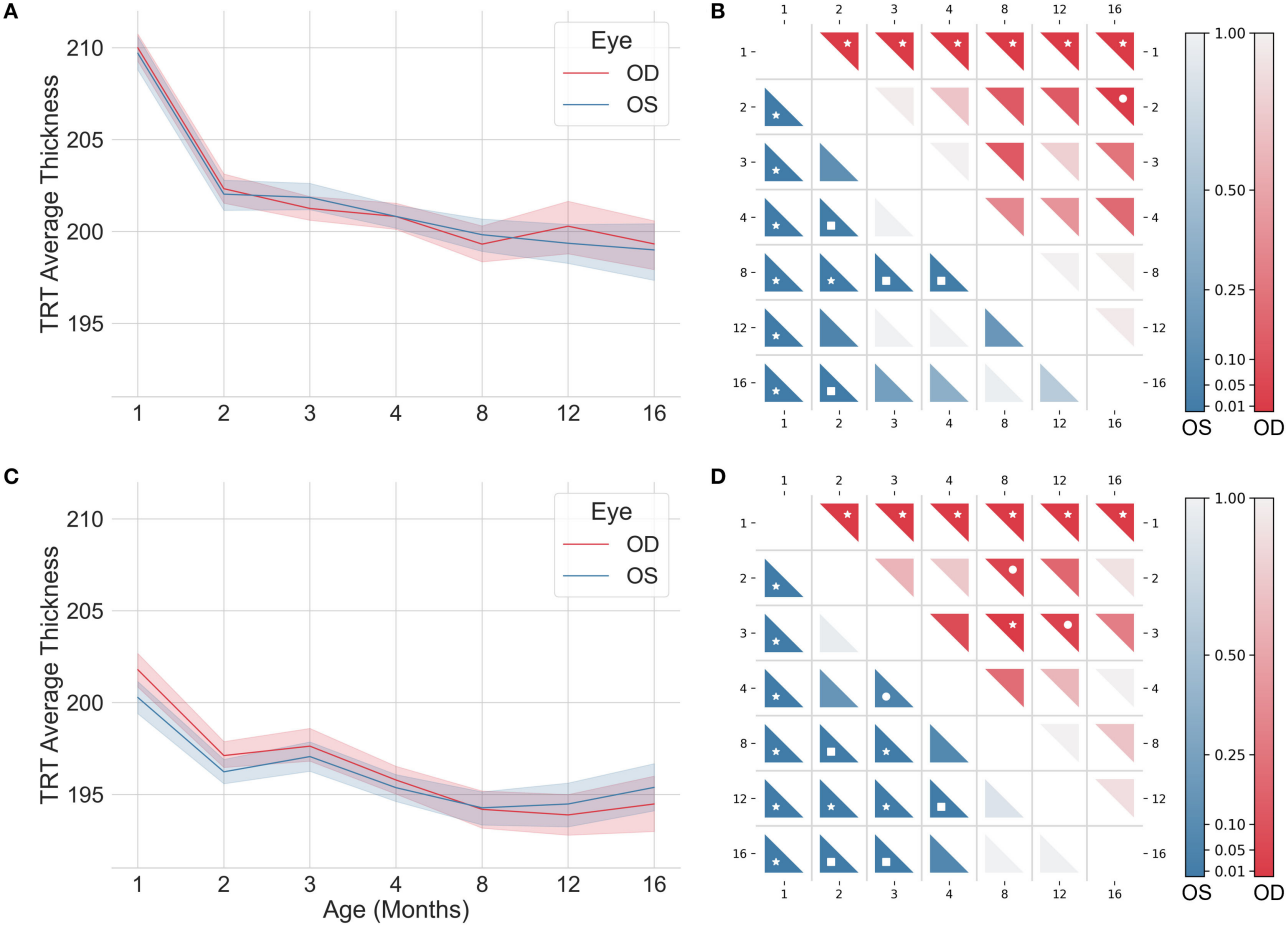
Longitudinal total retinal thickness of wild-type **(A, B)** and the triple transgenic Alzheimer's disease **(C, D)** mice for left (OS; blue) and right (OD; red) eyes. Color coded *p*-values for pairwise comparisons are shown in **(B)** and **(D)**. The color indicates the level of the *p*-value, as indicated by the color bars. *p*-values < 0.05 (•), < 0.01 (■), and < 0.001 (*).

Analysis of the thickness distributions over the timespan monitored in this study showed that the thickness of most retinal layers/layer aggregates are age dependent in both groups. Furthermore, the pattern of differences across age range differed between the two groups for all retinal layers. Longitudinal changes in TRT for WT and 3 × Tg-AD mice, differentiated by eye, are shown in Figures 5A, C, respectively. Corresponding pairwise comparisons, corrected for multiple comparisons, are shown in Figures 5B, D. Statistically significant differences were found between the TRT of 1 month-old mice and that of all other ages for both eyes in WT and 3 × Tg-AD mice. Supplementary Figure 1 through 8 show detailed data for each retinal layer/layer aggregate.

### 3.3. Normative thickness data

**Figure 6:**
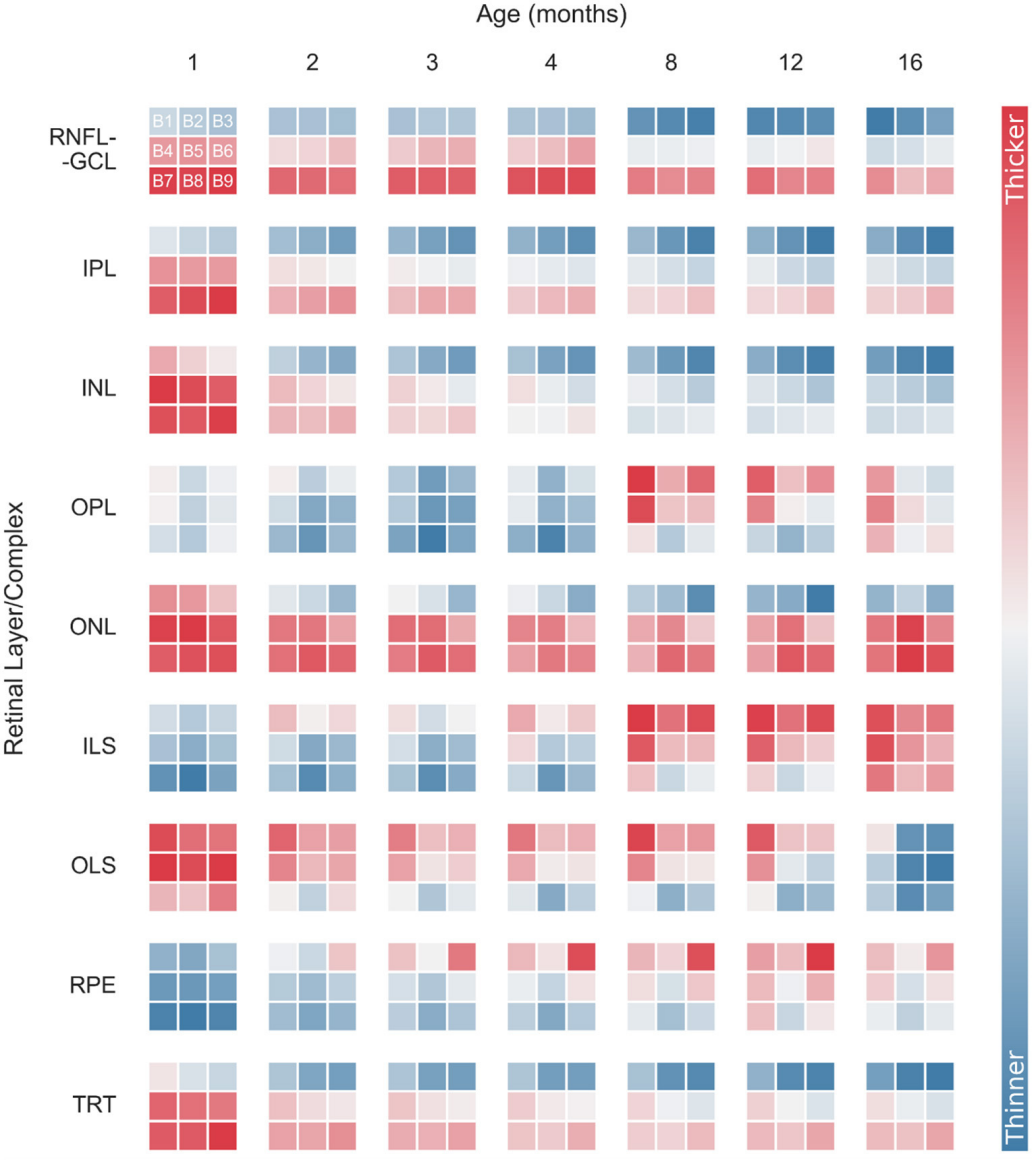
Normalized longitudinal mean retinal thickness variations for wild-type mice, differentiated by layer and block (B1–B9). Data were normalized for each retinal layer/layer aggregates. Normalized thickness is indicated by the color bar. RNFL-GCL, retinal nerve fiber layer and ganglion cell layer complex; IPL, inner plexiform layer; INL, inner nuclear layer; OPL, outer plexiform layer; ONL, outer nuclear layer; ILS, photoreceptor inner segments; OLS, photoreceptor outer segments; RPE, retinal pigment epithelium; TRT, total retina thickness.

Normative thickness maps were also generated by computing the average and standard deviation of 3 × 3 blocks of 170 × 170 points each. Figure 6 shows longitudinal variations in retinal thickness differentiated by layer and block for WT mice. For simplicity, the data were normalized for each retinal layer/layer aggregate. Data from both eyes were used to generate the thickness maps. Detailed longitudinal mean thickness values (and standard deviation) for each retinal layer of WT mice, discriminated by block and by eye, are shown in Supplementary Table 5 through 13.

As mentioned above, the temporal-nasal and superior-inferior TRT asymmetries are evident for all time points (Figure 6). The RNFL-GCL complex, IPL, INL, and ONL also appear thicker near the optic disc (B7–B9) and in the temporal side (B1, B4, and B7). However, the thickness of the OPL, ILS, OLS, and RPE increases with the distance to the optic disc. Moreover, these layers showed a more homogeneous thickness distribution between the temporal and nasal sides (Figure 6).

Monitoring thickness changes over time in smaller regions provides a better understanding of age-related retinal changes. In general, individual blocks follow the overall thickness trend observed while analyzing the total imaged area. However, there are some exceptions. For instance, the RPE shows an overall increase in thickness (Figures 3, 4). However, this increase is more pronounced in the superior and nasal sides. While the RPE of WT mice at 1 month of age is a homogeneous layer in thickness, the thickness values become more heterogeneous as the mice age (Figure 6 and Supplementary Table 12). For the ONL, although an overall decrease in layer thickness is observed, greater thickness changes are detected further away from the optic disc (Figure 6 and Supplementary Table 9).

Normative thickness maps can also be used to compute the probability that the retina of a given mouse has thickness values outside the normal range. Examples of these applications can be found in our previous publications (Ferreira et al., 2021; Bernardes et al., 2022).

## 4. Discussion

### 4.1. Animal models

Animal models of disease play a pivotal role in clinical research. Because of their relatively short lifespan, mice are ideal for aging research (Mitchell et al., 2015; Dutta and Sengupta, 2016), and are considered the animal model of choice for the studying of human aging (Mitchell et al., 2015). Mice models have already led to a better understanding of age-related neurochemical and behavioral changes (Davis and Squire, 1984; Rosenzweig and Barnes, 2003; Bishop et al., 2010). As mentioned above, the most widely used mouse inbred strain is C57BL/6J. It is used in several areas of research, including immunology, oncology, and longevity interventions. Recently, Yanai and Endo (2021) reported on how this mouse model relates to human aging. The authors characterized functional aging in male C57BL/6J mice using a multi-domain behavioral test battery. They observed a progressive decline in physical function beginning at 6 months of age. The decline in cognitive function began later in life, with significant deterioration observed at 22 months of age (Yanai and Endo, 2021). The relative age of onset of functional aging was also found to be lower in the C57BL/6J male mouse model than in humans (Yanai and Endo, 2021).

C57BL/6J mice are also widely used to study neurodegenerative diseases. Although there are no perfect *in vivo* models of human disease, mice are the ideal surrogate for studying specific aspects of pathology that are relevant to the human condition. Despite the differences in rodent and human biology, e.g., rodents lack the macula (the central region of the human retina) and red cones, they have more similarities than differences, and their retinas are organized in the same layers. As a result, rodent models can recapitulate critical elements of the disease process and are the most widely used animals to study the retina in vision research. The 3 × Tg-AD mouse model of familial AD, which harbors three genetic mutations, has been shown to develop age-related, progressive neuropathology, including senile plaques, mainly formed by amyloid beta (Aβ) peptide, and neurofibrillary tangles, mainly composed of hyperphosphorylated tau protein, characteristic of the human familial and sporadic of AD. Extracellular Aβ deposits are observed in the frontal cortex at 6 months of age and become more extensive by 12 months of age. Although tau pathology is not observed at 6 months, it is evident by 12 months (Oddo et al., 2003; Billings et al., 2005). Synaptic dysfunction occurs prior to the neuropathological hallmarks. Similarly, retention/retrieval deficits occur before plaques and tangles. Stover et al. (2015) observed that 6.5-month-old 3 × Tg-AD mice display learning and memory deficits. Moreover, we have previously reported that 4-, 8- and 16-month-old 3xTg-AD mice present impaired locomotor activity and recognition memory (Chiquita et al., 2019b). Concerning hippocampal volume, a substantial reduction was observed in 4-month-old mice that was exacerbated in older animals, with 8, 12, and 16 months of age (Chiquita et al., 2019b). This mouse model allows evaluation of the functional interactions that occur with concurrent Aβ and tau pathology, as well as the contribution to other pathological features of AD, such as neuroinflammation, cerebrovascular dysfunction, and cognitive deficits (Tai et al., 2021). However, the human tau mutant harbored by this mouse model causes frontotemporal dementia or other tauopathies in humans. Furthermore, tau protein is not mutated in Alzheimer's disease, and the distribution of tau pathology differs between Alzheimer's disease and frontotemporal dementia.

### 4.2. Normative longitudinal retinal thickness

Normative longitudinal retinal thickness data for WT mice are still lacking. Over the past years, our group has been working to fill these gaps. Recently, we reported on longitudinal normative retinal thicknesses for WT mice aged up to 4 months (Ferreira et al., 2021). Here, we continue the previous work by presenting a comprehensive characterization of healthy WT C57BL6/129S mice retinal thickness based on OCT imaging of the ocular fundus over 16 months. The effect of aging in eight retinal layers/layer aggregates was also evaluated to provide detailed normative data for the entire image area and nine ROIs. Along with the increase in the timespan covered, we also optimized the neural network model used for retinal layer segmentation (Bernardes et al., 2022) which increased the number of OCT scans meeting the segmentation quality criteria at all time points.

Direct comparison of retinal thickness with the literature is difficult due to differences in the OCT acquisition region, layer aggregation, and animal age. Nevertheless, TRT values are consistent with those reported in the literature (Ferguson et al., 2013, 2014; Berger et al., 2014; Kim et al., 2019; Ferdous et al., 2021; Ma et al., 2022). Ferguson et al. (2013) reported TRT values of 202.74 (4.85) μm for the superior retina of C57BL/6 WT mice, measured as the distance from the RNFL to the RPE superior to the optic disc, in 30 mice aged 3–5 months. These values are consistent with those measured in 3- [201.54 (2.46) μm] and 4-months-old [200.82 (2.48) μm] mice. Interestingly, the authors report a slightly thicker TRT for the temporal retina [205.18 (5.23)] when compared to the nasal retina [204.97 (6.71)] (Ferguson et al., 2013) supporting the observed TRT thickness nasal-temporal asymmetry. Kim et al. (2019) evaluated the retinal thickness longitudinally in a group of seven C57BL/6J WT mice aged 2 weeks to 2 months. The authors report a TRT of ~200 μm, with a decrease with age, which is consistent with our findings (Kim et al., 2019). Ferdous et al. (2021) also observed a significant decrease in retinal thickness with age in C57BL/6J WT mice. Here, the authors measured the retina of 4 different groups of WT mice of both sexes at ages ranging from 2 to 6 months (*N* = 13), 12–18 months (*N* = 12), 18–24 months (*N* = 9), and 24–32 months (*N* = 8) (Ferdous et al., 2021). More recently, Ma et al. (2022) reported a mean TRT of 238.5 (2.3) μm in C57BL/6J WT mice at 2 weeks of age. In mice aged 14 months (56 weeks), the TRT was 213.1 (3.2) μm. Five to six mice were included in each age group (Ma et al., 2022).

Although mean TRT values around 200 μm are consistently reported in the literature for WT mice, retinal layer/layer aggregate thicknesses show more discrepancies depending on age. Very few studies have compared retinal layer/layer aggregate thicknesses (Ferguson et al., 2013, 2014; Berger et al., 2014; Chiquita et al., 2019a; Ma et al., 2022) (Table 4). For ease of comparison, different layers and mice age groups were combined where appropriate to match the results reported by other groups. Chalam's group reported mean thickness values of 78.78 μm (*N* = 30) (Ferguson et al., 2013) and 68.08 μm (*N* = 8) (Ferguson et al., 2014) for the RNFL-GCL-IPL complex in mice with 3–5 months of age. Our results indicate a thinner RNFL-GCL-IPL complex, with values of 59.31 and 58.95 μm for WT mice at 3 and 4 months of age, respectively. Nevertheless, Ma et al. (2022) obtained an IPL thickness consistent with our results. For mice aged ~4 months (17 weeks) and 5 months (21 weeks), they report IPL thicknesses of 46.4 (2.4) and 46.1 (3.5) μm, respectively (Ma et al., 2022). Berger et al. (2014) also report an IPL thickness of ~50 μm in mice with ages ranging between 1.5 and 3 months. For the INL, our results are consistent with those reported by Ferguson et al. (2013) [27.82 (4.04) μm], Ferguson et al. (2014) [24.78 (0.6) μm], and those reported by Berger et al. (2014) (~25 μm). Thickness values for the INL-OPL aggregate reported by Chiquita et al. (2019a) for 4-months-old mice (*N* = 21) are also consistent with our findings. However, thicker INLs have also been reported, with values ranging from 33.6 (1) μm in mice aged 14 months (56 weeks) to 51.5 (1.5) μm in 2-week-old mice (Ma et al., 2022). The same was verified for ONL thickness values, where Ma et al. (2022) reported thickener ONL values [4 months: 75.6 (2.2) μm; 5 months: 72.7 (2.5) μm] than those reported in this study [3 months: 60.03 (1.12) μm; 4 months: 59.63 (0.97) μm] and those reported by Ferguson et al. (2013) [62.8 (6.23) μm], Berger et al. (2014) (~60 μm), Ferguson et al. (2014) [60.75 (0.70) μm], and Chiquita et al. (2019a) (~57 μm). Our OPL and the ILS-OLS/ILS-OLS-RPE aggregate thicknesses are also consistent with the literature (Ferguson et al., 2013, 2014; Chiquita et al., 2019a).

**Table 4:** Comparison of mean thickness of retinal layers/layer aggregates reported in the literature.

	**Ferguson et al. (2013)**	**Ferguson et al. (2014)**	**Berger et al. (2014)**	**Ma et al. (2022)**	**Chiquita et al. (2019a)**
Age	3–5 months	3–5 months	1.5–3 months	~4 and 5 months (17 and 21 weeks)	4 months
*N*	30	8	11	10–12	21
RNFL	78.78	68.08	–	9.9	–
GCL				–	≈57.5
IPL			≈50	46.25	
INL	27.82	24.78	≈25	43.65	≈37.5
OPL	19.22	15.80	≈12.5	–	
ONL	62.80	60.75	≈60	74.15	≈57
ILS	44.27	41.55	–	–	≈36
OLS			–	–	
RPE			–	–	–

RNFL, retinal nerve fiber layer; GCL, ganglion cell layer; IPL, inner plexiform layer; INL, inner nuclear layer; OPL, outer plexiform layer; ONL, outer nuclear layer; ILS, photoreceptor inner segments; OLS, photoreceptor outer segments; RPE, retinal pigment epithelium; N, number of animals.

### 4.3. AD-induced retinal thickness changes

Combined with the characterization of the WT mice retina and retinal layers/layer aggregates thickness with age, in this study we explored thickness changes in the AD model in age-matched transgenic mice. Overall, a progressive decrease in retinal thickness was observed with age in both WT and transgenic mice. A similar trend was also reported by Harper et al. (2020) for animals between 10 months (40 weeks) and 25 months of age (100 weeks) and by Chiquita et al. (2019a) for animal between 4 and 16 months of age. At all ages studied, we found that the retina of 3 × Tg-AD mice was thinner than that of age-matched WT mice. This is consistent with findings reported in the literature for animal models of AD (Chiquita et al., 2019a; Salobrar-García et al., 2021) and human patients (Marziani et al., 2013).

Considering AD-induced changes to individual layer/layer aggregate thickness, Chidlow et al. (2017) investigated changes in IPL, INL, OPL, and ONL thickness in the APP/PS1 mouse model of AD at 9–10 (*N* = 25) and 11–12 months of age (*N* = 10) using immunohistochemistry. However, unlike what we observed using retinal thicknesses based on OCT imaging of the ocular fundus, the authors did not find significant differences (Chidlow et al., 2017). The apparent contradictions with our results may be due to the different mouse model of AD used in the two studies. The APP/PS1 mouse model is a double transgenic mouse model with genes expressing mutant amyloid precursor protein and presenilin 1. In this study, we resorted to the triple transgenic mouse model, which, in addition to the mutant genes mentioned above, also expresses the mutant microtubule-associated protein tau. The different methods used to quantify retinal thickness may also help justify the differences observed.

Similar to our findings, Chiquita et al. (2019a) found significantly lower thicknesses in the GCL-IPL, INL-OPL, and ILS-OLS aggregates of male 3 × Tg-AD mice 4, 8, 12, and 16 months of age when compared to age-matched WT mice.

Recently, Song et al. (2020) reported a tendency for thinner OPL and RPE layers in female 3 × Tg-AD mice aged 15-16 months (*N* = 13 retinas) when compared to WT controls (*N* = 10 retinas). Although these changes did not reach statistical significance, this might be related to the smaller number of animals used. In contrast to our observation, the authors reported a significant decrease in RNFL thickness in the triple-transgenic AD animal model (Song et al., 2020). This may be explained by differences in the retinal layer segmentation process and the sex of the mice. Sex-bias in pathological phenotyping has been reported for the 3 × Tg-AD mouse model, with female mice showing a higher predisposition to develop both plaques and tangles (Javonillo et al., 2022). There is also some debate in the literature about the effect of AD on RNFL thickness. Although some groups have reported a significant decrease in the RNFL thickness in AD patients (Kesler et al., 2011; Marziani et al., 2013), in a large study of 414 cognitively healthy individuals and 324 patients meeting diagnostic criteria for AD found no significant changes (Sánchez et al., 2018).

## 5. Conclusion

This study provides a comprehensive normative database of retinal thickness in WT C57BL6/129S mice using OCT imaging. Our results demonstrate that retinal thickness is influenced by both age and imaging position relative to the optic disc. In addition to TRT, we report thickness changes in eight different layers/layer aggregates. Compared to age-matched WT mice, 3 × Tg-AD transgenic mice had lower overall retinal thickness and in multiple layers, except for the RNFL-GCL aggregate. Our findings are consistent with previous reports in the literature for animals up to 4 months of age and provide valuable information for future studies on retinal changes in animal models of neurodegenerative diseases such as AD. Additionally, the normative data for WT mice at 8, 12, and 16 months of age can serve as a reference for evaluating the effects of disease progression over extended periods of time, which has not been possible to date.

## Data availability statement

The raw data supporting the conclusions of this article will be made available by the authors, upon formal and reasonable request.

## Ethics statement

The animal study was reviewed and approved by Animal Welfare Committee of Coimbra Institute for Clinical and Biomedical Research.

## Author contributions

RB accounted for fundraising. RB, PG, and PS contributed to the conceptualization and study design. RB and PS contributed to the project administration. JM performed OCT scans. AB and PG performed data processing. AB and PS contributed to statistical analysis. AB, PG, PS, JM, MC-B, and RB performed the analysis and interpretation of data. AB, PG, PS, and RB completed an initial review and provided significant edits and additional content before review and approval by the other authors. All authors have read, commented on, and approved the manuscript.
