# Corrigendum: Normative mice retinal thickness: 16-month longitudinal characterization of wild-type mice and changes in a model of Alzheimer's disease

**DOI:** 10.3389/fnagi.2024.1423913

**Published:** 2024-05-10

**Authors:** Ana Batista, Pedro Guimarães, João Martins, Paula I. Moreira, António Francisco Ambrósio, Miguel Castelo-Branco, Pedro Serranho, Rui Bernardes

**Affiliations:** ^1^Coimbra Institute for Biomedical Imaging and Translational Research (CIBIT), Institute for Nuclear Sciences Applied to Health (ICNAS), University of Coimbra, Coimbra, Portugal; ^2^Coimbra Institute for Clinical and Biomedical Research (iCBR), Faculty of Medicine (FMUC), University of Coimbra, Coimbra, Portugal; ^3^Center for Innovative Biomedicine and Biotechnology (CIBB), University of Coimbra, Coimbra, Portugal; ^4^Clinical Academic Center of Coimbra (CACC), Faculty of Medicine (FMUC), University of Coimbra, Coimbra, Portugal; ^5^Laboratory of Physiology, Faculty of Medicine (FMUC), University of Coimbra, Coimbra, Portugal; ^6^Center for Neuroscience and Cell Biology (CNC), University of Coimbra, Coimbra, Portugal; ^7^Mathematics Section, Department of Sciences and Technology, Universidade Aberta, Lisbon, Portugal

**Keywords:** optical coherence tomography, retinal thickness, normative data, Alzheimer's disease, 3 × Tg-AD animal model

In the published article, there was an error in the Funding statement. The correct Funding statement appears below.

