# Supplementary material for: Normative mice retinal thickness: 16-month longitudinal characterization of wild-type mice and changes in a model of Alzheimer's disease
Source: Front Aging Neurosci. 2023 Apr 6;15:1161847. doi: 10.3389/fnagi.2023.1161847 (PMC10117679; doi:10.3389/fnagi.2023.1161847)
Supplement: Supplementary file 1 [file Data_Sheet_1.pdf]

Supplementary Material

Normative mice retinal thickness: 16-month longitudinal characterization of wild-type mice and changes in a model of Alzheimer's disease

Ana Batista,* Pedro Guimarães, Hugo Ferreira, João Martins, Paula I. Moreira, António Francisco Ambrósio, Miguel Castelo-Branco, Pedro Serranho, Rui Bernardes*

*** Correspondence:** Ana Batista: ana.batista@uc.pt; Rui Bernardes: rmbernardes@fmed.uc.pt

1 Supplementary Figures and Tables

1.1 Supplementary Figures

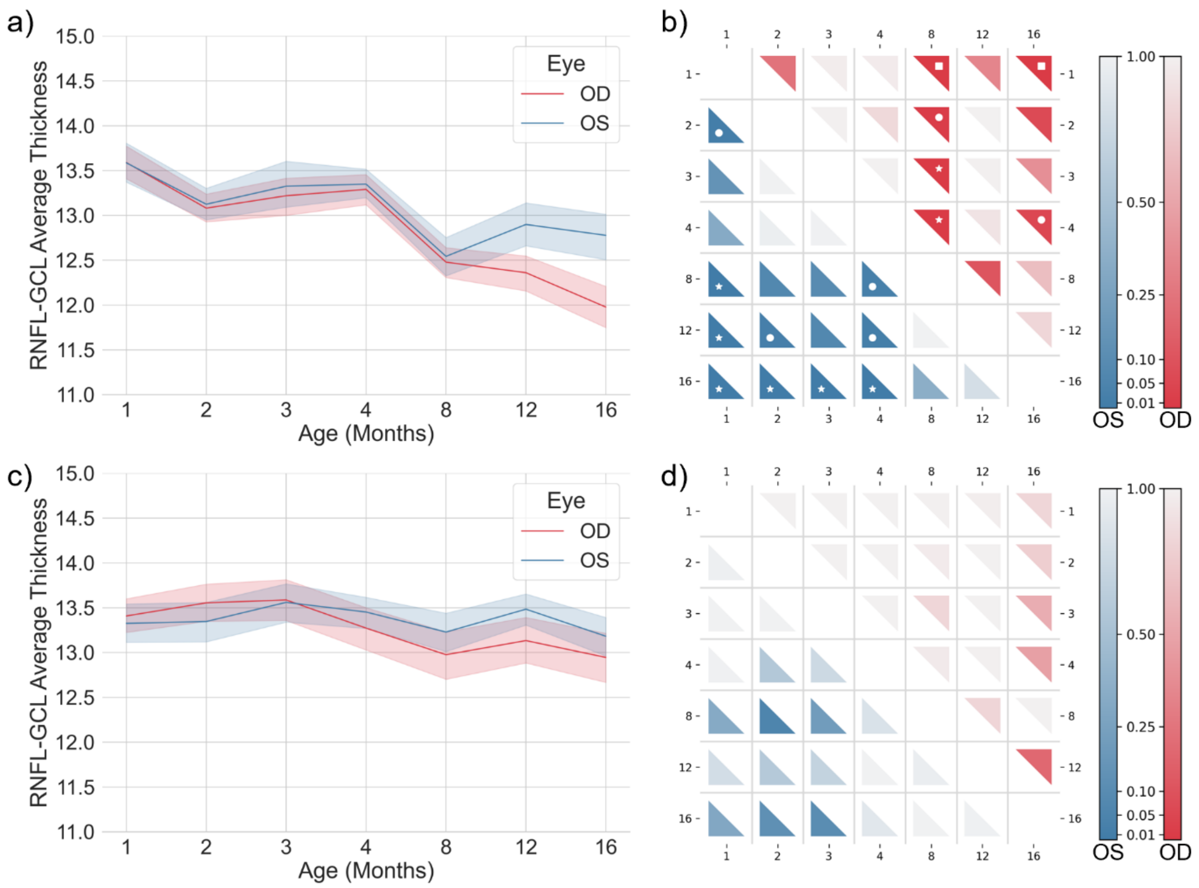

Supplementary Figure 1 Longitudinal retinal nerve fiber layer and ganglion cell layer complex (RNFL-GCL) thickness of wild-type (a, b) and the triple transgenic Alzheimer's disease (c, d) mice for left (OS; blue) and right (OD; red) eyes. Pair-wise comparisons are shown in b) and d). Color indicates the level of the *p*-value, as indicated by color bars. *p*-values < 0.05 (•), < 0.01 (▪), and < 0.001 (*).

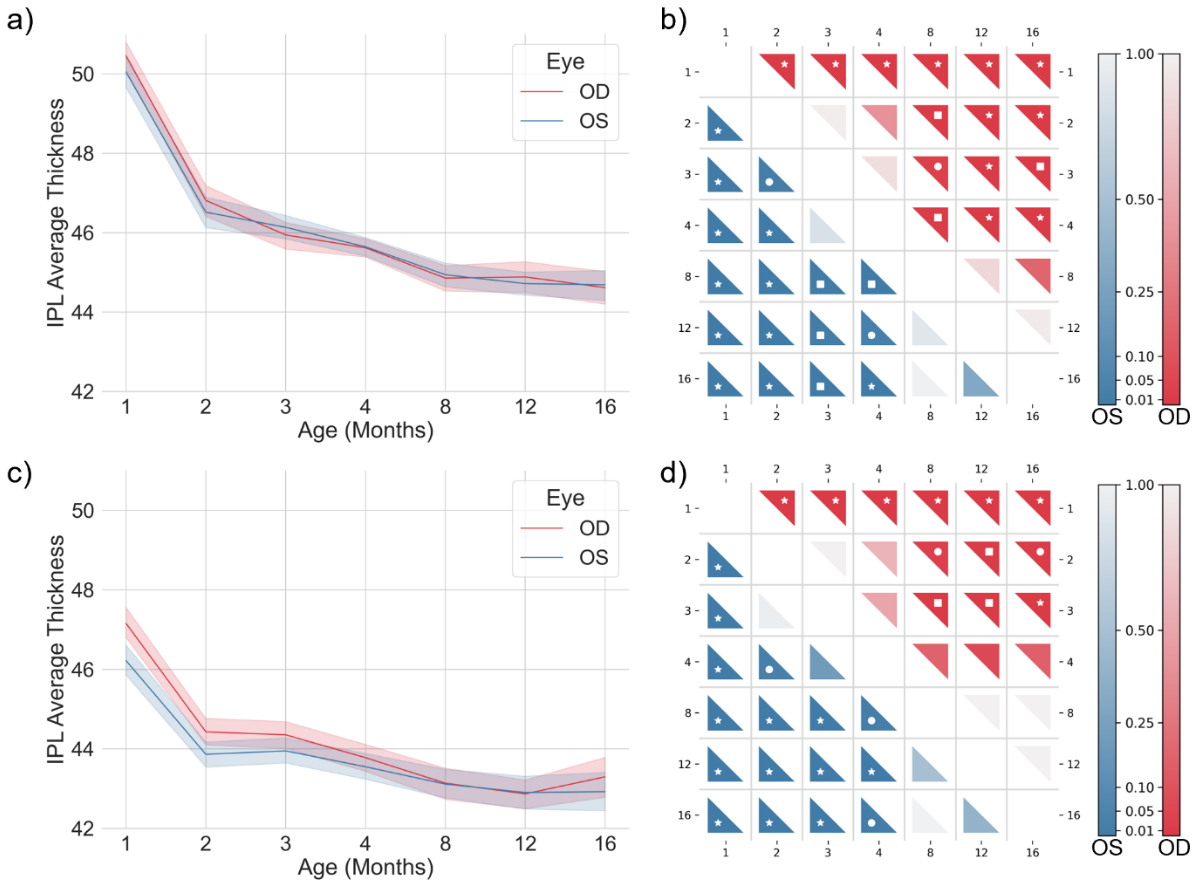

Supplementary Figure 2 Longitudinal inner plexiform layer (IPL) thickness of wild-type (a, b) and the triple transgenic Alzheimer's disease (c, d) mice for left (OS; blue) and right (OD; red) eyes. Pair-wise comparisons are shown in b) and d). Color indicates the level of the *p*-value, as indicated by color bars. *p*-values < 0.05 (•), < 0.01 (▪), and < 0.001 (*).

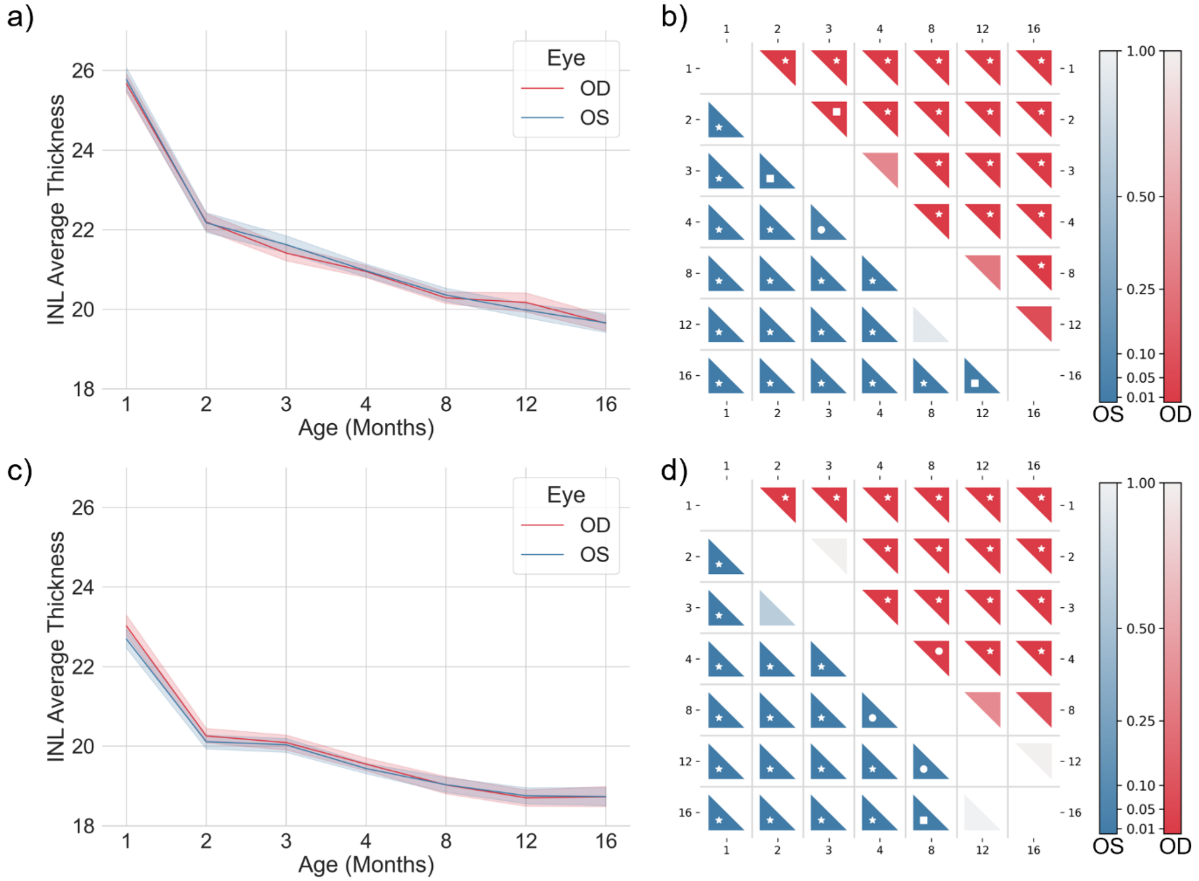

Supplementary Figure 3 Longitudinal inner nuclear layer (INL) thickness of wild-type (a, b) and the triple transgenic Alzheimer's disease (c, d) mice for left (OS; blue) and right (OD; red) eyes. Pair-wise comparisons are shown in b) and d). Color indicates the level of the *p*-value, as indicated by color bars. *p*-values < 0.05 (•), < 0.01 (▪), and < 0.001 (*).

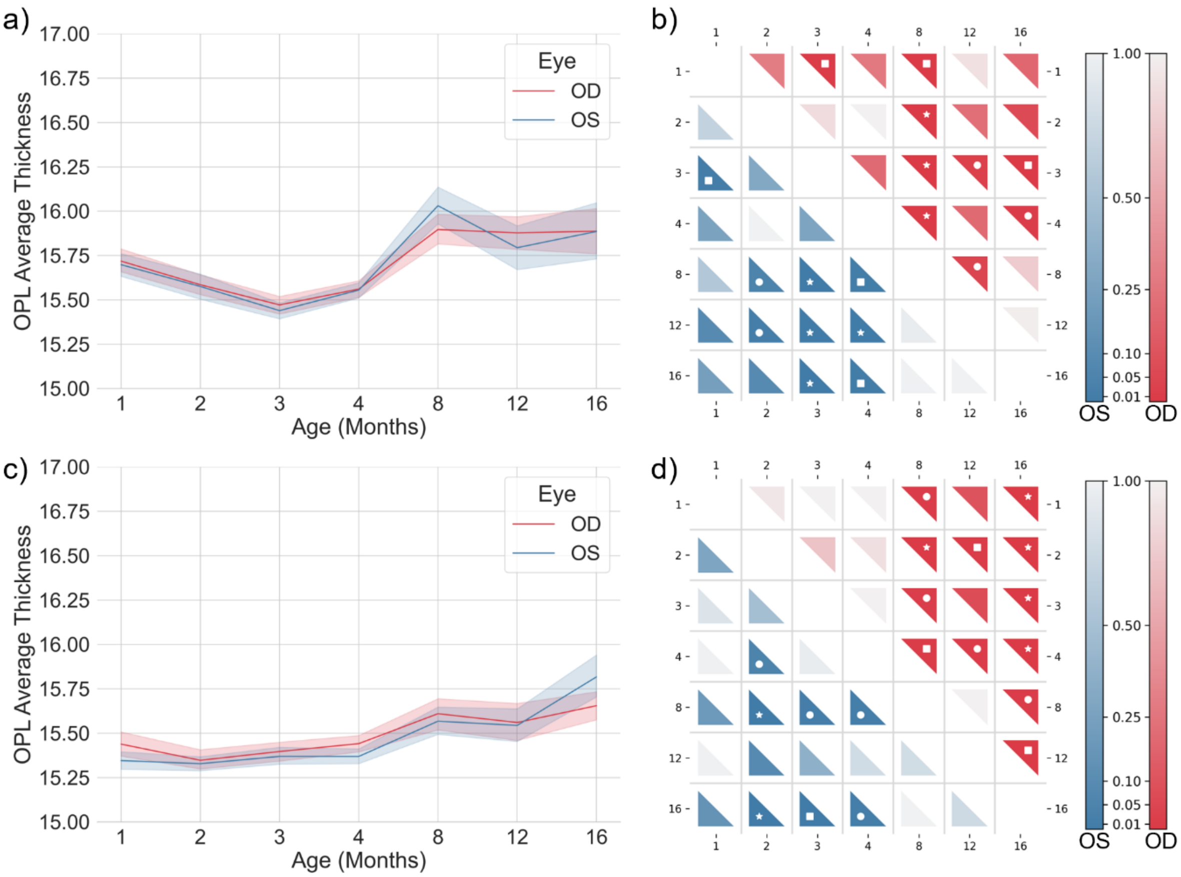

Supplementary Figure 4 Longitudinal outer plexiform layer (OPL) thickness of wild-type (a, b) and the triple transgenic Alzheimer's disease (c, d) mice for left (OS; blue) and right (OD; red) eyes. Pair-wise comparisons are shown in b) and d). Color indicates the level of the *p*-value, as indicated by color bars. *p*-values < 0.05 (•), < 0.01 (▪), and < 0.001 (*).

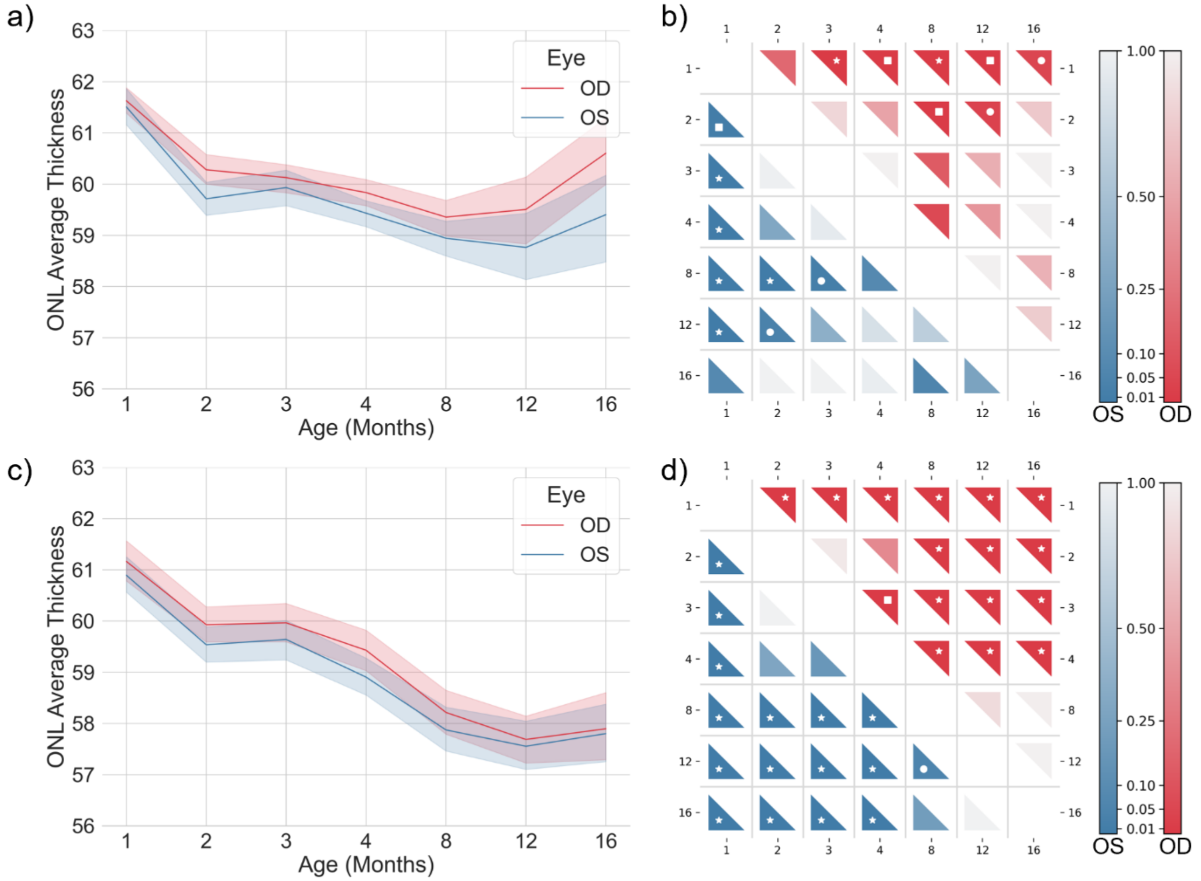

Supplementary Figure 5 Longitudinal outer nuclear layer (ONL) thickness of wild-type (a, b) and the triple transgenic Alzheimer's disease (c, d) mice for left (OS; blue) and right (OD; red) eyes. Pair-wise comparisons are shown in b) and d). Color indicates the level of the *p*-value, as indicated by color bars. *p*-values < 0.05 (•), < 0.01 (▪), and < 0.001 (*).

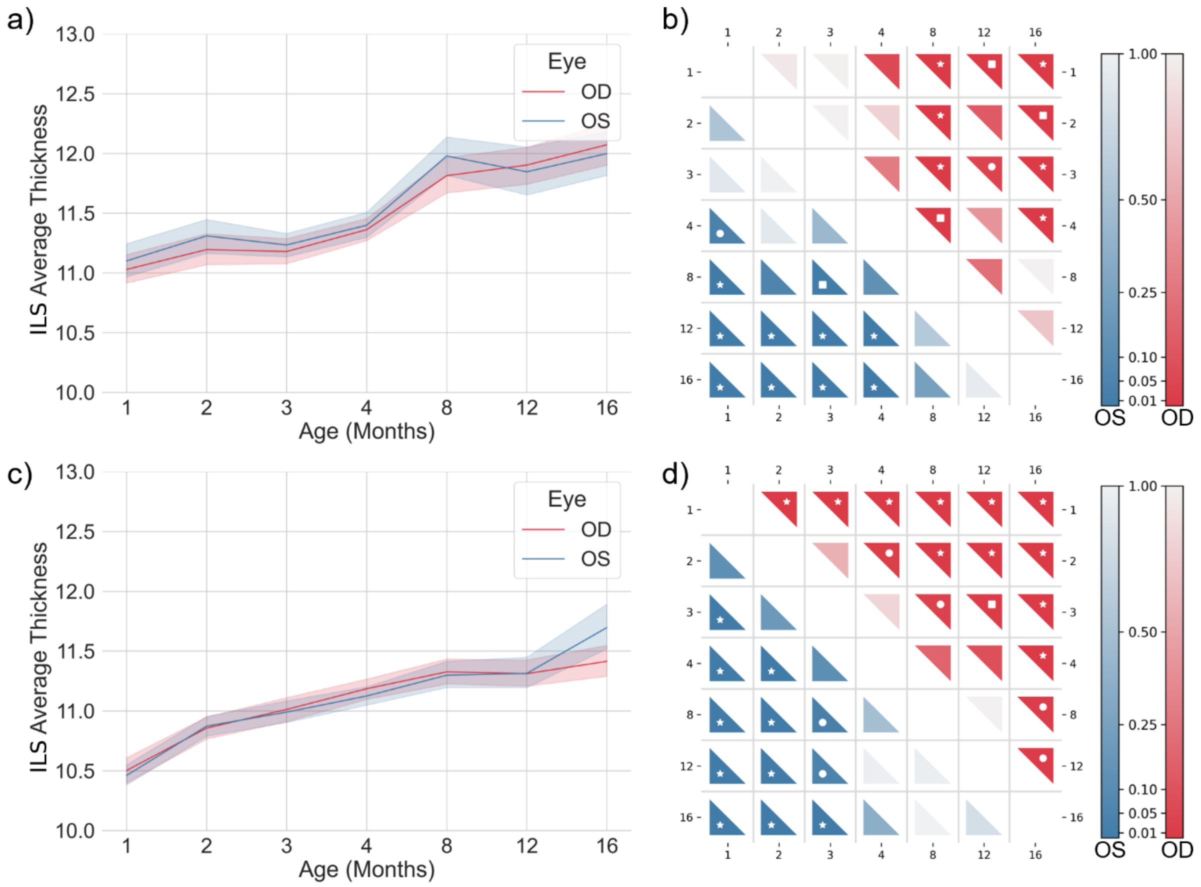

Supplementary Figure 6 Longitudinal photoreceptor inner segments (ILS) thickness of wild-type (a, b) and the triple transgenic Alzheimer's disease (c, d) mice for left (OS; blue) and right (OD; red) eyes. Pair-wise comparisons are shown in b) and d). Color indicates the level of the *p*-value, as indicated by color bars. *p*-values < 0.05 (•), < 0.01 (▪), and < 0.001 (*).

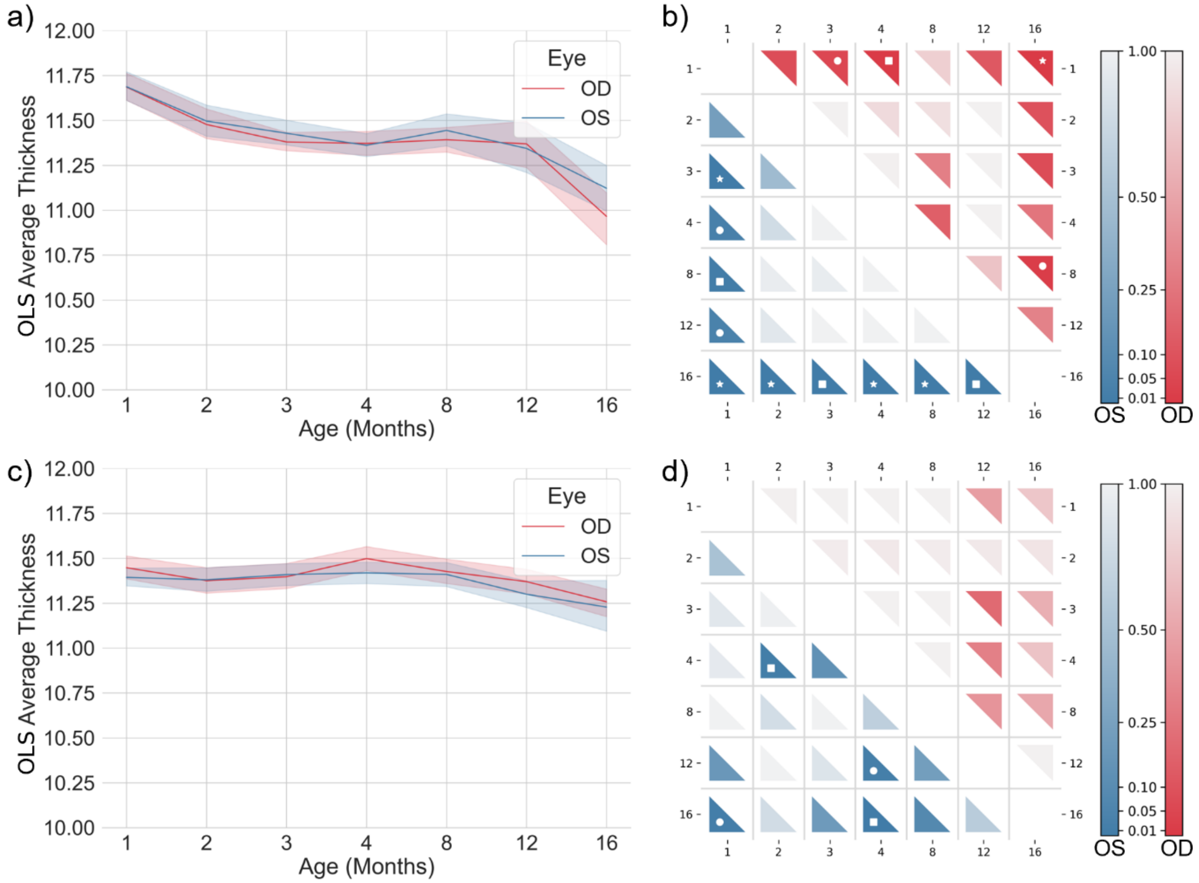

Supplementary Figure 7 Longitudinal photoreceptor outer segments (OLS) thickness of wild-type (a, b) and the triple transgenic Alzheimer's disease (c, d) mice for left (OS; blue) and right (OD; red) eyes. Pair-wise comparisons are shown in b) and d). Color indicates the level of the *p*-value, as indicated by color bars. *p*-values < 0.05 (•), < 0.01 (▪), and < 0.001 (*).

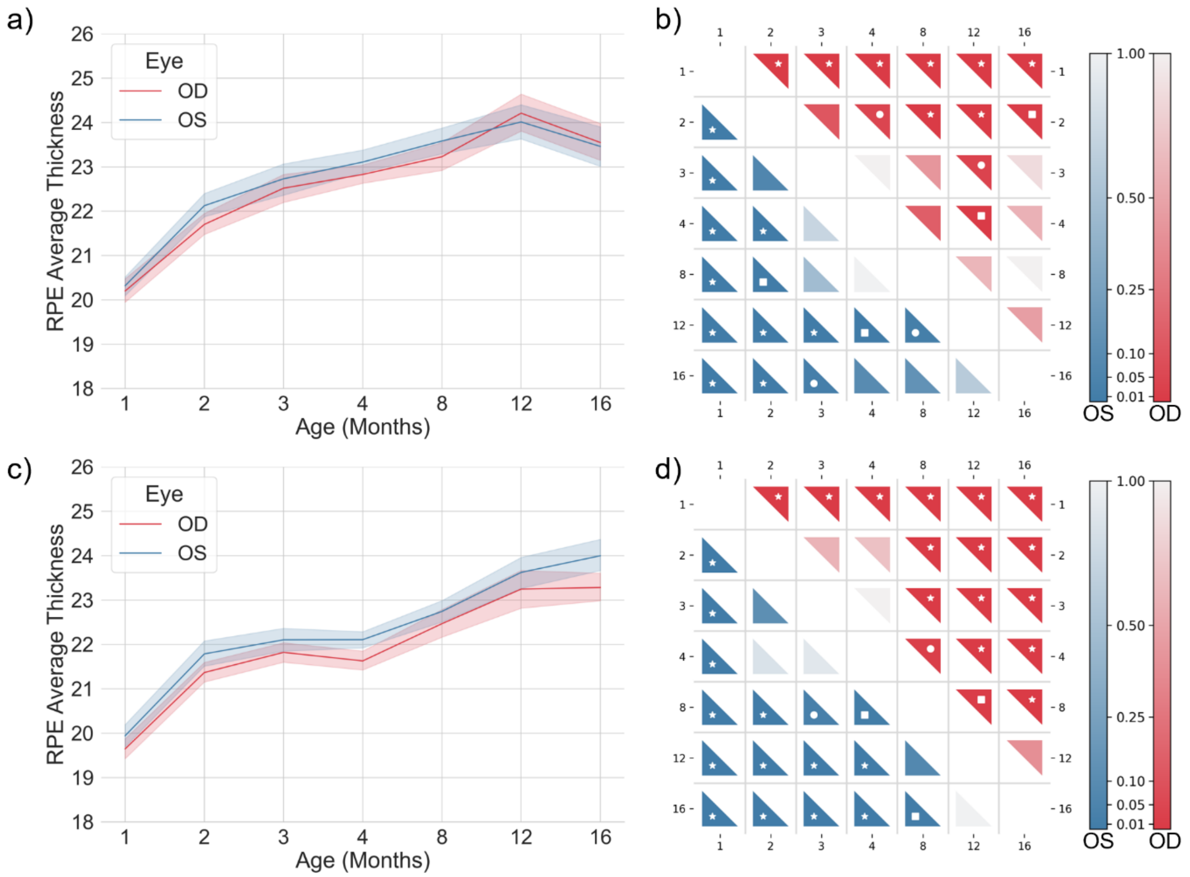

Supplementary Figure 8 Longitudinal retinal pigment epithelium (RPE) thickness of wild-type (a, b) and the triple transgenic Alzheimer's disease (c, d) mice for left (OS; blue) and right (OD; red) eyes. Pair-wise comparisons are shown in b) and d). Color indicates the level of the *p*-value, as indicated by color bars. *p*-values < 0.05 (•), < 0.01 (▪), and < 0.001 (*).

1.2 Supplementary Tables

Supplementary Table 1 Average percentage of regions of interest (ROIs) excluded from analysis for wild-type (WT) and the triple transgenic mouse model of Alzheimer's disease (3×Tg-AD) discriminated by block and by eye. For each eye, B1 to B9 are organized from left to right and top to bottom. OD and OS stand for oculus dexter and oculus sinister, respectively. Data are presented in %.

WT						3×Tg-AD					
OS			OD			OS			OD		
4.7	4.1	8.5	14.4	5.8	5.8	7.9	7.9	8.1	6.6	4.9	5.8
5.5	3.8	9.3	11.1	5.0	6.1	8.1	6.5	7.1	5.2	4.1	4.7
5.8	6.0	11.5	10.5	6.6	4.4	6.5	6.0	7.6	4.9	4.1	3.8

Supplementary Table 2 Longitudinal thickness values, in μm, for the left eye of wild-type (WT) and the triple transgenic Alzheimer's disease (3×Tg-AD) mice. Data are presented as mean (standard deviation).

	**Age (Months)**	1	2	3	4	8	12	16
**WT**	RNFL-GCL	13.59 (0.82)	13.12 (0.67)	13.33 (0.95)	13.35 (0.61)	12.54 (0.77)	12.90 (0.84)	12.78 (0.68)
	IPL	50.05 (1.33)	46.52 (1.43)	46.13 (1.09)	45.64 (0.86)	44.94 (1.05)	44.72 (0.96)	44.69 (1.08)
	INL	25.77 (1.08)	22.17 (0.89)	21.62 (0.80)	20.97 (0.59)	20.36 (0.59)	19.98 (0.66)	19.66 (0.65)
	OPL	15.70 (0.24)	15.58 (0.26)	15.44 (0.17)	15.56 (0.17)	16.03 (0.38)	15.79 (0.43)	15.89 (0.43)
	ONL	61.51 (1.28)	59.71 (1.18)	59.93 (1.22)	59.43 (0.97)	58.94 (1.24)	58.76 (2.29)	59.40 (2.36)
	ILS	11.10 (0.54)	11.31 (0.55)	11.23 (0.37)	11.40 (0.38)	11.98 (0.59)	11.85 (0.70)	12.00 (0.50)
	OLS	11.69 (0.31)	11.50 (0.31)	11.43 (0.26)	11.36 (0.24)	11.44 (0.33)	11.34 (0.45)	11.12 (0.35)
	RPE	20.32 (0.79)	22.12 (0.98)	22.73 (1.30)	23.11 (0.99)	23.58 (1.02)	24.01 (1.37)	23.46 (1.25)
	TRT	209.70 (3.31)	202.03 (3.03)	201.85 (2.53)	200.82 (2.45)	199.82 (3.14)	199.36 (3.70)	199.00 (4.15)
**3×Tg-AD**	RNFL-GCL	13.33 (0.81)	13.35 (0.82)	13.56 (0.84)	13.45 (0.65)	13.23 (0.76)	13.48 (0.59)	13.18 (0.70)
	IPL	46.22 (1.45)	43.87 (1.21)	43.95 (1.20)	43.55 (1.19)	43.12 (1.25)	42.90 (1.40)	42.93 (1.56)
	INL	22.69 (0.96)	20.11 (0.68)	20.04 (0.67)	19.44 (0.49)	19.03 (0.67)	18.76 (0.69)	18.74 (0.76)
	OPL	15.35 (0.17)	15.33 (0.14)	15.37 (0.19)	15.37 (0.16)	15.57 (0.26)	15.54 (0.31)	15.82 (0.39)
	ONL	60.90 (1.36)	59.54 (1.34)	59.64 (1.50)	58.91 (1.36)	57.87 (1.54)	57.56 (1.55)	57.80 (2.01)
	ILS	10.46 (0.32)	10.87 (0.32)	10.99 (0.34)	11.13 (0.29)	11.30 (0.39)	11.31 (0.43)	11.70 (0.66)
	OLS	11.39 (0.19)	11.38 (0.24)	11.41 (0.23)	11.42 (0.22)	11.41 (0.24)	11.30 (0.25)	11.23 (0.48)
	RPE	19.94 (1.00)	21.79 (1.07)	22.10 (0.97)	22.11 (0.69)	22.74 (0.87)	23.62 (1.18)	24.00 (1.19)
	TRT	200.28 (3.46)	196.23 (2.53)	197.06 (3.17)	195.37 (2.62)	194.27 (3.24)	194.48 (3.90)	195.39 (4.44)

RNFL-GCL – retinal nerve fiber layer and ganglion cell layer complex; IPL – inner plexiform layer; INL – inner nuclear layer; OPL – outer plexiform layer; ONL – outer nuclear layer; ILS – photoreceptor inner segments; OLS – photoreceptor outer segments, RPE – retinal pigment epithelium; TRT – total retina thickness.

Supplementary Table 3 Longitudinal thickness values, in μm, for the right eye of wild-type (WT) and the triple transgenic Alzheimer's disease (3×Tg-AD) mice. Data are presented as mean (standard deviation).

	**Age (Months)**	1	2	3	4	8	12	16
**WT**	RNFL-GCL	13.59 (0.71)	13.08 (0.59)	13.22 (0.79)	13.29 (0.64)	12.48 (0.65)	12.36 (0.68)	11.98 (0.69)
	IPL	50.46 (1.31)	46.81 (1.43)	45.94 (1.20)	45.62 (0.86)	44.86 (1.15)	44.89 (1.37)	44.62 (1.21)
	INL	25.67 (0.91)	22.19 (0.83)	21.41 (0.70)	20.95 (0.56)	20.29 (0.54)	20.17 (0.83)	19.65 (0.58)
	OPL	15.72 (0.25)	15.59 (0.21)	15.47 (0.18)	15.56 (0.17)	15.90 (0.31)	15.88 (0.33)	15.89 (0.40)
	ONL	61.63 (0.92)	60.28 (1.09)	60.13 (1.02)	59.84 (0.94)	59.36 (1.23)	59.51 (2.32)	60.60 (1.94)
	ILS	11.03 (0.47)	11.20 (0.49)	11.18 (0.38)	11.36 (0.34)	11.81 (0.52)	11.90 (0.55)	12.07 (0.51)
	OLS	11.69 (0.28)	11.48 (0.30)	11.38 (0.20)	11.37 (0.26)	11.39 (0.26)	11.37 (0.47)	10.97 (0.43)
	RPE	20.20 (1.02)	21.70 (0.90)	22.52 (1.18)	22.83 (0.79)	23.23 (1.17)	24.21 (1.51)	23.55 (1.20)
	TRT	210.00 (2.87)	202.33 (2.76)	201.25 (2.37)	200.82 (2.53)	199.31 (3.53)	200.29 (4.95)	199.32 (3.85)
**3×Tg – AD**	RNFL-GCL	13.41 (0.73)	13.55 (0.80)	13.59 (0.83)	13.27 (0.86)	12.98 (0.91)	13.13 (0.88)	12.95 (0.89)
	IPL	47.16 (1.45)	44.43 (1.28)	44.35 (1.24)	43.78 (1.24)	43.14 (1.44)	42.87 (1.23)	43.30 (1.57)
	INL	23.02 (0.96)	20.26 (0.67)	20.09 (0.70)	19.55 (0.58)	19.03 (0.77)	18.71 (0.65)	18.73 (0.75)
	OPL	15.44 (0.27)	15.35 (0.19)	15.40 (0.21)	15.44 (0.17)	15.61 (0.31)	15.56 (0.36)	15.65 (0.25)
	ONL	61.17 (1.43)	59.93 (1.29)	59.97 (1.38)	59.43 (1.45)	58.21 (1.50)	57.69 (1.53)	57.90 (2.01)
	ILS	10.50 (0.39)	10.86 0.34)	11.01 (0.37)	11.19 (0.31)	11.33 (0.38)	11.31 (0.37)	11.42 (0.40)
	OLS	11.45 (0.25)	11.38 (0.26)	11.40 (0.26)	11.50 (0.25)	11.43 (0.24)	11.37 (0.23)	11.26 (0.24)
	RPE	19.65 (0.87)	21.37 (0.84)	21.82 (0.76)	21.63 (0.75)	22.47 (1.04)	23.25 (1.44)	23.28 (1.02)
	TRT	201.79 (3.34)	197.12 (2.65)	197.63 (3.25)	195.79 (2.74)	194.19 (3.51)	193.89 (3.73)	194.48 (4.60)

RNFL-GCL – retinal nerve fiber layer and ganglion cell layer complex; IPL – inner plexiform layer; INL – inner nuclear layer; OPL – outer plexiform layer; ONL – outer nuclear layer; ILS – photoreceptor inner segments; OLS – photoreceptor outer segments, RPE – retinal pigment epithelium; TRT – total retina thickness.

Supplementary Table 4 Percentage of increase (↑) or decrease (↓) in layer thickness over the timespan covered for wild-type (WT) and the triple transgenic Alzheimer's Disease (3×Tg-AD) mice. Data presented as mean (standard deviation).

Retinal Layer	WT	3×Tg-AD
RNFL-GCL	↓8.8 (7.1)	↓2.1 (7.4)
IPL	↓11.3 (2.6)	↓7.8 (3.6)
INL	↓23.0 (2.9)	↓18.0 (4.6)
OPL	↑1.3 (2.6)	↑2.1 (2.7)
ONL	↓2.2 (3.5)	↓5.1 (2.8)
ILS	↑9.1 (5.3)	↑10.1 (6.4)
OLS	↓5.1 (4.1)	↓1.5 (3.8)
RPE	↑16.8 (8.5)	↑19.6 (7.6)
TRT	↓4.9 (1.9)	↓3.1 (2.6)

RNFL-GCL – retinal nerve fiber layer and ganglion cell layer complex; IPL – inner plexiform layer; INL – inner nuclear layer; OPL – outer plexiform layer; ONL – outer nuclear layer; ILS – photoreceptor inner segments; OLS – photoreceptor outer segments, RPE – retinal pigment epithelium; TRT – total retina thickness.

Supplementary Table 5 Normative longitudinal retinal nerve fiber layer and ganglion cell layer complex (RNFL-GCL) thickness maps for each block, for the right (OD) and left (OS) eyes separately, as well as thickness values obtained by combining both eyes' data (OD and OS) of WT mice. Data are represented in µm as mean (standard deviation).

	1 Month			2 Months			3 Months			4 Months			8 Months			12 Months			16 Months		
OS	12.24 (0.73)	12.07 (0.78)	11.84 (0.61)	12.04 (0.72)	12.06 (1.00)	11.90 (0.97)	12.08 (1.32)	12.06 (1.40)	11.82 (1.31)	12.01 (0.81)	11.98 (1.02)	11.67 (1.06)	11.12 (0.99)	11.02 (1.05)	10.68 (1.04)	11.09 (1.25)	11.36 (1.38)	11.22 (1.15)	11.13 (1.31)	11.49 (1.22)	11.18 (1.05)
	13.70 (1.00)	13.88 (1.16)	13.80 (1.00)	13.00 (0.66)	13.10 (0.65)	13.36 (0.91)	13.30 (1.14)	13.43 (0.86)	13.51 (0.90)	13.22 (0.57)	13.44 (0.67)	13.69 (0.75)	12.74 (1.04)	12.80 (0.97)	12.65 (0.93)	12.96 (1.19)	13.16 (1.24)	13.14 (1.00)	12.91 (0.90)	12.96 (1.03)	12.67 (1.16)
	14.89 (1.23)	14.82 (1.10)	14.92 (1.16)	14.30 (0.99)	14.29 (1.14)	14.26 (1.04)	14.59 (1.06)	14.54 (1.04)	14.62 (1.04)	14.61 (0.72)	14.65 (0.82)	14.87 (0.90)	14.11 (0.93)	13.86 (0.75)	14.10 (0.79)	14.52 (0.93)	14.16 (0.76)	14.35 (0.96)	14.29 (0.88)	13.75 (0.98)	13.95 (1.05)
OD	12.30 (0.60)	12.00 (0.68)	11.86 (0.76)	11.69 (0.79)	11.64 (1.08)	11.70 (1.21)	11.66 (1.31)	11.88 (1.27)	12.07 (1.11)	11.78 (0.85)	11.74 (1.06)	11.77 (1.17)	10.89 (1.02)	10.65 (1.08)	10.60 (1.08)	10.49 (0.98)	10.40 (0.98)	10.61 (0.94)	9.94 (1.12)	10.23 (1.03)	11.34 (1.51)
	13.90 (0.79)	13.87 (1.15)	13.76 (0.95)	13.08 (0.60)	13.10 (0.64)	13.38 (0.72)	13.14 (0.84)	13.50 (0.76)	13.60 (0.81)	13.16 (0.67)	13.31 (0.78)	13.77 (0.89)	12.55 (1.02)	12.51 (0.95)	12.72 (0.79)	12.34 (1.11)	12.30 (1.12)	12.65 (0.97)	11.78 (1.35)	11.81 (1.06)	12.60 (0.80)
	14.85 (0.94)	14.80 (0.97)	14.96 (1.12)	14.49 (0.76)	14.44 (0.73)	14.30 (0.88)	14.45 (0.80)	14.47 (0.80)	14.36 (0.88)	14.66 (0.62)	14.79 (0.70)	14.62 (0.72)	14.19 (0.61)	14.05 (0.61)	14.05 (0.59)	14.10 (0.98)	13.91 (0.79)	13.86 (0.82)	13.64 (1.13)	13.07 (0.89)	13.26 (0.86)
OS+OD	12.27 (0.66)	12.03 (0.73)	11.85 (0.69)	11.87 (0.77)	11.85 (1.06)	11.80 (1.09)	11.86 (1.33)	11.97 (1.33)	11.95 (1.22)	11.89 (0.83)	11.86 (1.04)	11.72 (1.11)	11.00 (1.00)	10.83 (1.08)	10.64 (1.05)	10.77 (1.15)	10.84 (1.27)	10.90 (1.08)	10.56 (1.36)	10.91 (1.29)	11.27 (1.31)
	13.80 (0.90)	13.87 (1.15)	13.78 (0.97)	13.04 (0.63)	13.10 (0.64)	13.37 (0.81)	13.22 (1.00)	13.47 (0.81)	13.56 (0.85)	13.19 (0.62)	13.38 (0.73)	13.73 (0.82)	12.64 (1.03)	12.65 (0.96)	12.69 (0.86)	12.64 (1.19)	12.73 (1.25)	12.89 (1.01)	12.31 (1.28)	12.40 (1.19)	12.63 (0.99)
	14.87 (1.09)	14.81 (1.03)	14.94 (1.13)	14.39 (0.88)	14.36 (0.95)	14.28 (0.95)	14.52 (0.93)	14.51 (0.92)	14.49 (0.97)	14.64 (0.67)	14.72 (0.76)	14.74 (0.82)	14.15 (0.79)	13.96 (0.69)	14.08 (0.70)	14.31 (0.98)	14.03 (0.78)	14.11 (0.93)	13.96 (1.06)	13.38 (0.99)	13.61 (1.02)

Supplementary Table 6 Normative longitudinal inner plexiform layer (IPL) thickness maps for each block, for the right (OD) and left (OS) eyes separately, as well as thickness values obtained by combining both eyes' data (OD and OS) of WT mice. Data are represented in µm as mean (standard deviation).

	1 Month			2 Months			3 Months			4 Months			8 Months			12 Months			16 Months		
**OS**	45.82	44.76	43.89	43.27	41.94	40.76	42.93	41.34	40.28	42.63	41.31	39.81	42.65	40.70	39.11	42.06	40.39	38.32	42.28	39.73	37.50
	(1.96)	(2.17)	(1.76)	(2.21)	(1.67)	(1.88)	(1.47)	(1.70)	(1.63)	(1.60)	(1.27)	(1.32)	(1.63)	(1.74)	(1.84)	(1.70)	(1.45)	(1.40)	(2.92)	(2.45)	(1.50)
	51.21	50.78	50.65	47.80	47.10	46.60	47.41	46.71	46.70	46.80	46.49	45.89	46.32	45.73	44.89	46.27	45.24	44.49	46.66	45.15	44.84
	(1.60)	(2.05)	(2.11)	(1.87)	(1.70)	(1.96)	(1.39)	(1.32)	(1.61)	(1.33)	(1.14)	(1.44)	(1.42)	(1.47)	(1.50)	(1.50)	(1.34)	(1.59)	(1.71)	(1.41)	(1.68)
	53.63	54.19	55.33	49.81	50.27	51.40	49.49	49.79	50.71	48.72	49.12	50.05	48.04	48.07	49.08	47.99	48.00	49.40	48.42	48.45	50.06
	(1.93)	(2.13)	(2.27)	(1.94)	(1.82)	(2.05)	(1.60)	(2.02)	(2.18)	(1.29)	(1.30)	(1.77)	(1.41)	(1.26)	(1.44)	(1.27)	(1.15)	(1.56)	(1.45)	(1.52)	(1.83)
**OD**	46.26	44.93	44.28	43.06	42.08	41.01	42.19	40.95	39.93	42.14	40.56	39.77	42.82	40.04	38.56	42.27	39.82	38.48	41.50	39.40	39.23
	(2.10)	(1.90)	(1.99)	(2.19)	(1.85)	(1.93)	(1.92)	(1.90)	(1.84)	(1.51)	(1.39)	(1.53)	(2.03)	(1.61)	(1.65)	(2.52)	(1.83)	(1.87)	(1.84)	(3.02)	(4.92)
	51.49	51.22	51.34	47.89	47.74	47.16	47.06	46.93	46.13	46.72	46.19	46.16	46.30	45.26	44.68	46.57	44.97	44.38	45.61	45.14	44.53
	(2.01)	(2.16)	(2.01)	(1.94)	(1.92)	(2.16)	(1.52)	(1.73)	(1.73)	(1.24)	(1.43)	(1.84)	(1.49)	(1.67)	(1.63)	(1.61)	(1.76)	(1.87)	(1.57)	(1.92)	(2.22)
	54.02	54.99	55.58	50.14	51.19	51.49	49.39	50.92	50.04	48.87	50.04	50.09	48.20	48.53	49.32	48.41	48.60	49.62	48.51	49.01	49.88
	(1.92)	(2.22)	(2.01)	(2.14)	(1.96)	(1.71)	(1.67)	(1.88)	(1.85)	(1.54)	(1.58)	(1.31)	(1.73)	(1.69)	(1.45)	(1.91)	(1.89)	(1.62)	(2.31)	(2.19)	(1.61)
**OS+OD**	46.04	44.85	44.09	43.17	42.01	40.89	42.55	41.14	40.10	42.39	40.93	39.79	42.73	40.37	38.83	42.17	40.08	38.41	41.90	39.58	38.44
	(2.04)	(2.03)	(1.88)	(2.19)	(1.75)	(1.90)	(1.75)	(1.81)	(1.74)	(1.57)	(1.38)	(1.42)	(1.83)	(1.70)	(1.76)	(2.16)	(1.68)	(1.66)	(2.47)	(2.71)	(3.83)
	51.35	51.01	51.00	47.84	47.43	46.88	47.24	46.82	46.41	46.76	46.34	46.02	46.31	45.49	44.79	46.42	45.10	44.43	46.10	45.14	44.69
	(1.82)	(2.11)	(2.08)	(1.90)	(1.83)	(2.07)	(1.46)	(1.54)	(1.69)	(1.28)	(1.30)	(1.65)	(1.45)	(1.58)	(1.56)	(1.55)	(1.56)	(1.73)	(1.71)	(1.66)	(1.95)
	53.83	54.60	55.46	49.97	50.74	51.45	49.44	50.36	50.37	48.79	49.58	50.07	48.12	48.30	49.20	48.20	48.30	49.51	48.47	48.75	49.97
	(1.93)	(2.20)	(2.14)	(2.04)	(1.94)	(1.88)	(1.63)	(2.02)	(2.04)	(1.42)	(1.51)	(1.55)	(1.57)	(1.50)	(1.44)	(1.63)	(1.59)	(1.58)	(1.92)	(1.92)	(1.71)

Supplementary Table 7 Normative longitudinal inner nuclear layer (INL) thickness maps for each block, for the right (OD) and left (OS) eyes separately, as well as thickness values obtained by combining both eyes' data (OD and OS) of WT mice. Data are represented in µm as mean (standard deviation).

	1 Month			2 Months			3 Months			4 Months			8 Months			12 Months			16 Months		
**OS**	24.11	23.06	22.45	20.58	19.41	18.86	20.41	19.11	18.37	19.92	18.56	17.92	19.55	18.17	17.33	18.87	17.58	16.86	18.33	17.25	16.47
	(1.46)	(1.36)	(1.29)	(1.33)	(1.11)	(0.89)	(1.46)	(1.11)	(0.77)	(1.03)	(0.82)	(0.56)	(1.14)	(0.98)	(0.70)	(1.04)	(0.81)	(0.58)	(1.09)	(1.31)	(0.63)
	27.41	27.02	26.60	23.66	23.04	22.43	23.08	22.57	21.94	22.66	21.97	21.32	21.95	21.34	20.50	21.37	20.89	20.09	20.95	20.47	19.97
	(1.29)	(1.23)	(1.24)	(1.19)	(1.19)	(1.05)	(1.02)	(0.98)	(0.83)	(0.84)	(0.76)	(0.72)	(0.88)	(0.86)	(0.74)	(0.92)	(1.08)	(0.90)	(0.78)	(0.83)	(0.80)
	26.96	26.68	27.37	23.79	23.78	24.00	22.96	22.90	23.24	22.04	21.92	22.40	21.30	21.50	21.69	21.00	21.42	21.65	20.88	21.08	21.36
	(1.15)	(1.32)	(1.31)	(0.84)	(0.85)	(0.86)	(0.94)	(0.88)	(0.95)	(0.71)	(0.70)	(0.79)	(0.83)	(0.74)	(0.68)	(0.84)	(0.81)	(0.71)	(0.80)	(0.89)	(0.76)
**OD**	24.33	23.13	22.35	20.62	19.35	18.77	19.71	18.59	18.03	19.80	18.43	17.78	19.63	17.99	17.16	19.04	17.55	16.91	18.18	17.12	16.96
	(1.36)	(1.13)	(1.11)	(1.09)	(0.92)	(0.75)	(1.30)	(0.92)	(0.65)	(0.98)	(0.77)	(0.58)	(0.94)	(0.73)	(0.71)	(1.24)	(0.88)	(0.69)	(0.74)	(1.45)	(1.68)
	27.56	26.89	26.32	23.82	22.95	22.45	23.09	22.20	21.55	22.69	21.76	20.96	21.91	21.08	20.12	21.66	20.94	20.01	20.89	20.33	19.63
	(1.06)	(1.04)	(1.19)	(1.01)	(1.01)	(1.09)	(1.01)	(0.85)	(0.73)	(0.79)	(0.70)	(0.61)	(0.70)	(0.83)	(0.75)	(0.94)	(1.14)	(1.03)	(1.15)	(0.90)	(0.81)
	26.74	26.46	27.29	23.90	23.63	24.17	23.17	22.94	23.51	22.22	22.15	22.73	21.32	21.51	21.80	21.45	21.65	21.88	21.12	21.21	21.50
	(1.18)	(1.11)	(0.89)	(0.97)	(0.98)	(1.02)	(0.80)	(0.79)	(0.79)	(0.72)	(0.82)	(0.78)	(0.64)	(0.70)	(0.61)	(0.88)	(0.97)	(0.88)	(1.14)	(0.90)	(0.64)
**OS+OD**	24.22	23.10	22.40	20.60	19.38	18.82	20.04	18.84	18.20	19.86	18.50	17.85	19.59	18.08	17.24	18.96	17.57	16.89	18.26	17.19	16.74
	(1.40)	(1.24)	(1.20)	(1.21)	(1.02)	(0.82)	(1.42)	(1.05)	(0.73)	(1.00)	(0.79)	(0.58)	(1.04)	(0.86)	(0.71)	(1.15)	(0.84)	(0.64)	(0.93)	(1.37)	(1.32)
	27.49	26.95	26.46	23.74	22.99	22.44	23.08	22.38	21.74	22.67	21.86	21.14	21.93	21.21	20.31	21.52	20.92	20.05	20.92	20.40	19.80
	(1.18)	(1.13)	(1.22)	(1.10)	(1.10)	(1.06)	(1.01)	(0.93)	(0.80)	(0.81)	(0.73)	(0.69)	(0.79)	(0.85)	(0.76)	(0.93)	(1.10)	(0.96)	(0.99)	(0.86)	(0.81)
	26.85	26.57	27.33	23.85	23.71	24.09	23.07	22.92	23.38	22.13	22.04	22.56	21.31	21.50	21.75	21.23	21.54	21.77	21.00	21.15	21.43
	(1.16)	(1.22)	(1.11)	(0.90)	(0.92)	(0.94)	(0.88)	(0.83)	(0.88)	(0.72)	(0.77)	(0.80)	(0.74)	(0.72)	(0.64)	(0.89)	(0.90)	(0.81)	(0.99)	(0.89)	(0.70)

Supplementary Table 8 Normative longitudinal outer plexiform layer (OPL) thickness maps for each block, for the right (OD) and left (OS) eyes separately, as well as thickness values obtained by combining both eyes' data (OD and OS) of WT mice. Data are represented in µm as mean (standard deviation).

	1 Month			2 Months			3 Months			4 Months			8 Months			12 Months			16 Months		
**OS**	15.82	15.66	15.74	15.79	15.60	15.75	15.59	15.38	15.50	15.74	15.49	15.70	16.27	15.97	16.24	16.06	15.81	16.05	16.02	15.74	15.81
	(0.41)	(0.30)	(0.31)	(0.52)	(0.50)	(0.50)	(0.28)	(0.28)	(0.39)	(0.23)	(0.25)	(0.31)	(0.51)	(0.48)	(0.52)	(0.67)	(0.69)	(0.81)	(0.65)	(0.62)	(0.51)
	15.76	15.60	15.70	15.67	15.46	15.49	15.60	15.40	15.44	15.75	15.49	15.56	16.34	15.96	16.03	16.04	15.77	15.74	16.02	15.81	15.77
	(0.27)	(0.23)	(0.24)	(0.29)	(0.23)	(0.20)	(0.25)	(0.18)	(0.20)	(0.23)	(0.21)	(0.21)	(0.53)	(0.36)	(0.42)	(0.55)	(0.45)	(0.42)	(0.43)	(0.35)	(0.47)
	15.67	15.59	15.74	15.51	15.38	15.54	15.41	15.24	15.44	15.48	15.28	15.50	15.88	15.62	15.81	15.56	15.48	15.62	15.90	15.70	15.85
	(0.35)	(0.43)	(0.32)	(0.20)	(0.24)	(0.21)	(0.17)	(0.17)	(0.19)	(0.22)	(0.18)	(0.20)	(0.45)	(0.26)	(0.35)	(0.31)	(0.27)	(0.28)	(0.50)	(0.39)	(0.41)
**OD**	15.77	15.66	15.78	15.79	15.63	15.74	15.61	15.43	15.56	15.74	15.52	15.69	16.30	15.96	16.07	16.28	15.99	16.04	16.02	15.71	15.57
	(0.33)	(0.30)	(0.32)	(0.35)	(0.41)	(0.45)	(0.33)	(0.34)	(0.40)	(0.23)	(0.23)	(0.29)	(0.45)	(0.43)	(0.53)	(0.68)	(0.50)	(0.54)	(0.54)	(0.54)	(0.52)
	15.79	15.65	15.76	15.68	15.46	15.52	15.61	15.39	15.43	15.73	15.50	15.53	16.13	15.86	15.81	16.13	15.81	15.74	16.14	15.88	15.70
	(0.28)	(0.25)	(0.25)	(0.24)	(0.20)	(0.23)	(0.22)	(0.19)	(0.20)	(0.22)	(0.20)	(0.21)	(0.39)	(0.35)	(0.37)	(0.42)	(0.31)	(0.41)	(0.45)	(0.58)	(0.38)
	15.70	15.61	15.77	15.56	15.39	15.52	15.48	15.31	15.47	15.50	15.33	15.50	15.77	15.58	15.65	15.74	15.54	15.60	15.99	15.81	15.82
	(0.29)	(0.32)	(0.32)	(0.21)	(0.19)	(0.22)	(0.16)	(0.17)	(0.18)	(0.16)	(0.18)	(0.19)	(0.25)	(0.19)	(0.22)	(0.31)	(0.23)	(0.30)	(0.38)	(0.30)	(0.24)
**OS+OD**	15.79	15.66	15.76	15.79	15.62	15.75	15.60	15.41	15.53	15.74	15.50	15.70	16.28	15.97	16.15	16.18	15.91	16.05	16.02	15.73	15.68
	(0.37)	(0.30)	(0.32)	(0.44)	(0.46)	(0.48)	(0.31)	(0.31)	(0.39)	(0.23)	(0.24)	(0.30)	(0.48)	(0.45)	(0.53)	(0.68)	(0.60)	(0.67)	(0.60)	(0.57)	(0.53)
	15.78	15.63	15.73	15.68	15.46	15.51	15.60	15.39	15.43	15.74	15.50	15.55	16.23	15.90	15.92	16.08	15.79	15.74	16.08	15.84	15.73
	(0.27)	(0.24)	(0.24)	(0.26)	(0.21)	(0.21)	(0.23)	(0.18)	(0.20)	(0.22)	(0.20)	(0.21)	(0.48)	(0.36)	(0.41)	(0.49)	(0.39)	(0.41)	(0.44)	(0.47)	(0.43)
	15.69	15.60	15.76	15.53	15.38	15.53	15.44	15.27	15.45	15.49	15.30	15.50	15.82	15.60	15.73	15.65	15.51	15.61	15.95	15.76	15.83
	(0.32)	(0.38)	(0.32)	(0.20)	(0.22)	(0.22)	(0.17)	(0.17)	(0.19)	(0.19)	(0.18)	(0.19)	(0.37)	(0.23)	(0.30)	(0.32)	(0.25)	(0.29)	(0.44)	(0.35)	(0.34)

Supplementary Table 9 Normative longitudinal outer inner nuclear layer (ONL) thickness maps for each block, for the right (OD) and left (OS) eyes separately, as well as thickness values obtained by combining both eyes' data (OD and OS) of WT mice. Data are represented in µm as mean (standard deviation).

	1 Month			2 Months			3 Months			4 Months			8 Months			12 Months			16 Months		
OS	60.53 (1.74)	60.42 (1.75)	59.47 (1.74)	57.89 (1.69)	57.41 (1.57)	56.31 (1.70)	58.87 (1.81)	58.21 (1.71)	56.64 (1.84)	58.42 (1.45)	57.51 (1.33)	56.07 (1.48)	57.21 (1.74)	56.48 (1.64)	54.83 (1.44)	56.15 (2.27)	55.74 (2.05)	54.04 (2.17)	56.07 (2.37)	57.16 (2.70)	55.55 (1.82)
	62.33 (1.43)	62.56 (1.39)	61.94 (1.48)	61.01 (1.26)	61.03 (1.33)	59.98 (1.28)	61.08 (1.32)	61.20 (1.38)	59.96 (1.35)	60.83 (1.18)	60.97 (1.32)	59.62 (1.28)	60.09 (1.78)	60.59 (1.84)	59.11 (1.86)	60.26 (2.97)	61.08 (3.16)	58.66 (2.35)	61.27 (3.62)	62.33 (3.07)	59.62 (2.87)
	61.94 (1.10)	62.04 (1.22)	62.01 (1.26)	61.25 (1.31)	61.68 (1.32)	61.18 (1.14)	60.88 (1.07)	61.69 (2.12)	61.02 (1.36)	60.19 (1.08)	60.87 (1.01)	60.41 (1.03)	60.31 (2.33)	61.64 (2.26)	60.48 (1.79)	60.65 (3.17)	62.06 (3.43)	60.41 (2.19)	61.76 (3.49)	62.64 (3.25)	60.99 (2.86)
OD	60.92 (1.33)	60.68 (1.18)	59.76 (1.35)	58.56 (1.42)	57.98 (1.30)	56.81 (1.30)	58.30 (1.54)	57.79 (1.41)	56.47 (1.40)	58.48 (1.25)	57.79 (1.16)	56.32 (1.18)	57.39 (1.50)	56.94 (1.67)	55.43 (1.75)	56.84 (1.66)	56.54 (2.42)	54.97 (2.20)	56.84 (1.66)	57.86 (2.15)	56.71 (2.30)
	62.55 (1.23)	62.65 (1.06)	61.93 (1.13)	61.48 (1.27)	61.48 (1.30)	60.61 (1.33)	61.86 (3.25)	61.76 (3.13)	60.31 (1.03)	61.08 (1.14)	61.26 (1.07)	60.00 (1.12)	60.27 (1.30)	61.15 (1.62)	59.78 (1.86)	60.30 (2.58)	61.68 (3.26)	60.53 (3.36)	61.25 (2.88)	62.48 (2.76)	62.17 (2.94)
	61.73 (1.05)	62.15 (0.87)	62.29 (0.89)	61.53 (1.19)	62.18 (1.16)	62.03 (1.15)	61.41 (1.09)	62.07 (1.11)	61.91 (1.12)	60.52 (1.18)	61.53 (1.17)	61.54 (1.21)	59.65 (1.29)	61.55 (1.80)	61.95 (1.94)	60.14 (2.26)	61.78 (2.66)	62.78 (3.07)	60.93 (2.50)	62.44 (2.46)	63.28 (2.76)
OS+OD	60.73 (1.55)	60.55 (1.48)	59.62 (1.56)	58.22 (1.59)	57.69 (1.46)	56.56 (1.53)	58.57 (1.69)	57.99 (1.57)	56.55 (1.62)	58.45 (1.35)	57.65 (1.25)	56.20 (1.34)	57.30 (1.62)	56.72 (1.66)	55.13 (1.62)	56.51 (1.99)	56.17 (2.28)	54.54 (2.22)	56.44 (2.08)	57.49 (2.46)	56.18 (2.16)
	62.44 (1.33)	62.61 (1.23)	61.94 (1.31)	61.25 (1.28)	61.26 (1.33)	60.30 (1.34)	61.47 (2.51)	61.48 (2.44)	60.13 (1.20)	60.95 (1.16)	61.12 (1.21)	59.81 (1.21)	60.18 (1.55)	60.88 (1.74)	59.45 (1.88)	60.28 (2.77)	61.39 (3.21)	59.61 (3.04)	61.26 (3.22)	62.40 (2.90)	60.88 (3.16)
	61.83 (1.08)	62.10 (1.05)	62.15 (1.09)	61.39 (1.26)	61.93 (1.26)	61.61 (1.22)	61.15 (1.10)	61.88 (1.70)	61.47 (1.32)	60.36 (1.14)	61.20 (1.14)	60.97 (1.25)	59.99 (1.91)	61.59 (2.03)	61.22 (2.00)	60.39 (2.75)	61.92 (3.06)	61.60 (2.91)	61.34 (3.04)	62.54 (2.83)	62.12 (3.02)

Supplementary Table 10 Normative longitudinal of the photoreceptor inner segments (ILS) thickness maps for each block, for the right (OD) and left (OS) eyes separately, as well as thickness values obtained by combining both eyes' data (OD and OS) of WT mice. Data are represented in µm as mean (standard deviation).

	1 Month			2 Months			3 Months			4 Months			8 Months			12 Months			16 Months		
OS	11.42 (0.62)	11.27 (0.62)	11.38 (0.56)	11.83 (0.82)	11.62 (0.81)	11.74 (0.71)	11.60 (0.50)	11.38 (0.61)	11.54 (0.60)	11.85 (0.55)	11.56 (0.51)	11.72 (0.47)	12.36 (0.73)	12.15 (0.71)	12.41 (0.73)	12.14 (0.95)	12.00 (0.99)	12.32 (1.15)	12.21 (0.76)	12.03 (0.62)	12.42 (0.87)
	11.16 (0.60)	11.03 (0.58)	11.19 (0.51)	11.32 (0.63)	11.03 (0.62)	11.20 (0.51)	11.38 (0.53)	11.07 (0.51)	11.23 (0.41)	11.60 (0.56)	11.24 (0.53)	11.35 (0.44)	12.25 (0.77)	11.81 (0.63)	11.93 (0.59)	12.02 (0.82)	11.77 (0.86)	11.75 (0.73)	12.10 (0.61)	11.85 (0.64)	11.78 (0.52)
	10.77 (0.69)	10.69 (0.74)	11.02 (0.61)	11.07 (0.49)	10.77 (0.56)	11.14 (0.48)	11.09 (0.45)	10.79 (0.47)	11.05 (0.35)	11.22 (0.42)	10.85 (0.39)	11.20 (0.33)	11.67 (0.56)	11.35 (0.48)	11.60 (0.49)	11.48 (0.54)	11.28 (0.52)	11.52 (0.49)	11.99 (0.53)	11.73 (0.54)	11.91 (0.45)
OD	11.27 (0.46)	11.14 (0.51)	11.23 (0.50)	11.68 (0.64)	11.41 (0.72)	11.51 (0.75)	11.60 (0.54)	11.33 (0.61)	11.46 (0.66)	11.84 (0.44)	11.53 (0.46)	11.66 (0.43)	12.35 (0.58)	12.02 (0.59)	12.12 (0.72)	12.48 (0.69)	12.17 (0.72)	12.23 (0.78)	12.28 (0.63)	11.95 (0.62)	11.78 (0.69)
	11.15 (0.51)	10.99 (0.55)	11.10 (0.53)	11.35 (0.50)	10.94 (0.52)	10.98 (0.55)	11.35 (0.40)	10.97 (0.49)	11.00 (0.51)	11.66 (0.39)	11.18 (0.48)	11.17 (0.44)	12.16 (0.60)	11.71 (0.61)	11.61 (0.69)	12.31 (0.67)	11.77 (0.63)	11.61 (0.67)	12.39 (0.63)	12.01 (1.03)	11.82 (0.53)
	10.86 (0.55)	10.64 (0.57)	10.87 (0.60)	11.21 (0.52)	10.76 (0.54)	10.92 (0.59)	11.20 (0.33)	10.78 (0.39)	10.95 (0.41)	11.39 (0.34)	10.84 (0.37)	10.98 (0.41)	11.79 (0.49)	11.28 (0.42)	11.33 (0.51)	11.85 (0.51)	11.33 (0.44)	11.44 (0.52)	12.14 (0.53)	11.81 (0.44)	11.92 (0.46)
OS+OD	11.35 (0.54)	11.21 (0.57)	11.30 (0.53)	11.75 (0.74)	11.52 (0.77)	11.63 (0.73)	11.60 (0.52)	11.35 (0.61)	11.50 (0.63)	11.84 (0.49)	11.55 (0.49)	11.69 (0.45)	12.35 (0.65)	12.09 (0.65)	12.26 (0.73)	12.32 (0.84)	12.09 (0.85)	12.27 (0.97)	12.25 (0.69)	11.99 (0.61)	12.07 (0.83)
	11.16 (0.56)	11.01 (0.56)	11.15 (0.52)	11.34 (0.57)	10.99 (0.57)	11.09 (0.54)	11.36 (0.47)	11.02 (0.50)	11.12 (0.48)	11.63 (0.48)	11.21 (0.50)	11.26 (0.45)	12.21 (0.69)	11.76 (0.62)	11.77 (0.66)	12.17 (0.76)	11.77 (0.75)	11.68 (0.70)	12.25 (0.63)	11.93 (0.85)	11.80 (0.52)
	10.82 (0.62)	10.66 (0.66)	10.94 (0.60)	11.14 (0.51)	10.77 (0.55)	11.03 (0.55)	11.15 (0.39)	10.79 (0.43)	11.00 (0.38)	11.31 (0.39)	10.85 (0.38)	11.09 (0.39)	11.73 (0.53)	11.31 (0.45)	11.46 (0.52)	11.67 (0.55)	11.31 (0.48)	11.48 (0.51)	12.07 (0.53)	11.77 (0.49)	11.91 (0.45)

Supplementary Table 11 Normative longitudinal of the photoreceptor outer segments (OLS) thickness maps for each block, for the right (OD) and left (OS) eyes separately, as well as thickness values obtained by combining both eyes' data (OD and OS) of WT mice. Data are represented in µm as mean (standard deviation).

	1 Month			2 Months			3 Months			4 Months			8 Months			12 Months			16 Months		
**OS**	11.78 (0.29)	11.68 (0.29)	11.68 (0.27)	11.71 (0.42)	11.58 (0.44)	11.60 (0.43)	11.66 (0.29)	11.50 (0.30)	11.58 (0.30)	11.64 (0.35)	11.48 (0.32)	11.55 (0.31)	11.80 (0.40)	11.65 (0.43)	11.72 (0.45)	11.59 (0.49)	11.41 (0.53)	11.49 (0.81)	11.35 (0.36)	10.94 (0.50)	10.95 (0.51)
	11.82 (0.36)	11.76 (0.33)	11.83 (0.29)	11.58 (0.36)	11.49 (0.35)	11.58 (0.28)	11.54 (0.34)	11.37 (0.29)	11.50 (0.26)	11.48 (0.36)	11.34 (0.36)	11.43 (0.28)	11.60 (0.39)	11.42 (0.39)	11.43 (0.36)	11.45 (0.64)	11.26 (0.68)	11.26 (0.55)	11.09 (0.54)	10.87 (0.71)	10.95 (0.53)
	11.49 (0.43)	11.45 (0.41)	11.65 (0.34)	11.28 (0.29)	11.14 (0.30)	11.43 (0.29)	11.28 (0.37)	11.10 (0.36)	11.33 (0.30)	11.17 (0.27)	10.96 (0.27)	11.21 (0.25)	11.19 (0.43)	10.95 (0.39)	11.11 (0.42)	11.24 (0.63)	10.98 (0.50)	11.13 (0.37)	11.03 (0.66)	10.83 (0.61)	10.99 (0.57)
**OD**	11.77 (0.24)	11.68 (0.26)	11.66 (0.26)	11.71 (0.33)	11.49 (0.33)	11.49 (0.36)	11.62 (0.31)	11.42 (0.30)	11.41 (0.31)	11.68 (0.36)	11.46 (0.35)	11.44 (0.34)	11.80 (0.33)	11.44 (0.35)	11.42 (0.41)	11.87 (0.40)	11.49 (0.60)	11.41 (0.54)	11.38 (0.41)	10.88 (0.55)	10.85 (0.49)
	11.83 (0.32)	11.79 (0.31)	11.84 (0.30)	11.66 (0.30)	11.48 (0.30)	11.49 (0.31)	11.54 (0.21)	11.34 (0.25)	11.33 (0.24)	11.56 (0.27)	11.34 (0.34)	11.30 (0.30)	11.63 (0.30)	11.31 (0.39)	11.27 (0.37)	11.73 (0.52)	11.31 (0.72)	11.09 (0.61)	11.23 (0.57)	10.83 (0.82)	10.67 (0.51)
	11.50 (0.36)	11.46 (0.35)	11.65 (0.34)	11.39 (0.39)	11.22 (0.40)	11.35 (0.40)	11.36 (0.24)	11.15 (0.26)	11.24 (0.26)	11.36 (0.23)	11.06 (0.25)	11.14 (0.30)	11.42 (0.29)	11.11 (0.36)	11.15 (0.38)	11.41 (0.56)	11.09 (0.50)	11.03 (0.57)	11.26 (0.68)	10.93 (0.56)	10.95 (0.66)
**OS+OD**	11.78 (0.26)	11.68 (0.27)	11.67 (0.26)	11.71 (0.37)	11.54 (0.39)	11.55 (0.40)	11.64 (0.30)	11.46 (0.30)	11.50 (0.32)	11.66 (0.35)	11.47 (0.34)	11.50 (0.33)	11.80 (0.36)	11.54 (0.40)	11.57 (0.46)	11.74 (0.46)	11.45 (0.57)	11.45 (0.67)	11.36 (0.38)	10.91 (0.52)	10.89 (0.49)
	11.83 (0.34)	11.78 (0.32)	11.83 (0.30)	11.62 (0.33)	11.48 (0.32)	11.53 (0.29)	11.54 (0.28)	11.36 (0.27)	11.42 (0.26)	11.52 (0.32)	11.34 (0.35)	11.36 (0.30)	11.62 (0.35)	11.36 (0.39)	11.35 (0.37)	11.59 (0.60)	11.28 (0.70)	11.18 (0.59)	11.16 (0.56)	10.85 (0.76)	10.81 (0.54)
	11.49 (0.40)	11.45 (0.38)	11.65 (0.34)	11.33 (0.35)	11.18 (0.36)	11.39 (0.35)	11.32 (0.31)	11.13 (0.31)	11.28 (0.29)	11.27 (0.27)	11.01 (0.26)	11.17 (0.28)	11.31 (0.38)	11.03 (0.38)	11.13 (0.40)	11.33 (0.60)	11.03 (0.50)	11.08 (0.48)	11.15 (0.67)	10.88 (0.58)	10.97 (0.61)

Supplementary Table 12 Normative longitudinal retinal pigment epithelium (RPE) thickness maps for each block, for the right (OD) and left (OS) eyes separately, as well as thickness values obtained by combining both eyes' data (OD and OS) of WT mice. Data are represented in µm as mean (standard deviation).

	1 Month			2 Months			3 Months			4 Months			8 Months			12 Months			16 Months		
OS	21.04 (1.24)	20.84 (1.15)	21.66 (1.31)	23.23 (1.46)	22.51 (1.21)	24.61 (2.12)	24.23 (1.97)	23.32 (1.95)	25.77 (3.17)	24.37 (1.46)	23.58 (1.22)	26.70 (1.82)	24.50 (1.56)	23.96 (1.40)	26.51 (2.16)	24.99 (2.12)	24.26 (1.81)	26.70 (2.32)	24.31 (1.84)	23.22 (1.57)	25.09 (1.66)
	20.18 (0.82)	20.21 (0.88)	20.55 (0.88)	21.77 (0.76)	21.41 (0.85)	22.33 (1.40)	22.49 (1.35)	21.78 (0.95)	23.02 (1.54)	22.88 (1.33)	22.11 (1.18)	23.97 (1.49)	23.54 (1.21)	22.65 (0.89)	24.40 (1.47)	23.83 (1.82)	22.83 (1.80)	24.58 (1.99)	23.35 (1.80)	22.29 (1.53)	23.64 (1.61)
	19.48 (1.00)	19.29 (0.92)	19.60 (0.87)	21.28 (0.70)	20.67 (0.68)	21.30 (0.95)	21.86 (1.16)	20.83 (0.71)	21.57 (0.96)	21.85 (1.01)	20.57 (0.80)	21.94 (1.12)	22.66 (1.39)	21.46 (1.17)	22.45 (1.06)	23.72 (1.55)	21.84 (1.31)	23.26 (1.45)	22.68 (1.67)	21.73 (1.15)	22.54 (1.33)
OD	20.94 (1.59)	20.50 (1.22)	21.31 (1.97)	22.71 (1.31)	21.88 (1.05)	23.30 (1.99)	23.86 (2.07)	22.81 (1.77)	25.23 (2.65)	24.12 (1.35)	23.18 (1.12)	26.12 (2.00)	24.10 (1.21)	23.39 (1.28)	26.18 (2.37)	24.52 (2.09)	23.98 (1.71)	26.91 (2.22)	23.99 (1.63)	23.20 (1.46)	24.81 (1.99)
	20.23 (1.03)	20.16 (0.83)	20.28 (1.09)	21.80 (0.85)	21.18 (0.69)	21.55 (1.11)	22.42 (1.29)	21.59 (0.89)	22.52 (1.32)	22.88 (0.99)	22.05 (0.87)	22.77 (1.21)	23.42 (1.35)	22.33 (1.04)	23.44 (1.69)	24.55 (1.69)	23.14 (1.33)	24.22 (1.70)	24.19 (1.96)	22.59 (1.66)	23.20 (1.48)
	19.51 (1.02)	19.26 (0.90)	19.60 (0.95)	21.33 (0.85)	20.56 (0.73)	20.97 (0.74)	21.92 (1.22)	20.82 (0.73)	21.63 (1.24)	21.97 (0.78)	20.84 (0.67)	21.54 (0.89)	22.87 (1.50)	21.38 (0.93)	22.03 (1.06)	24.48 (2.17)	22.45 (1.45)	23.34 (1.83)	23.09 (1.50)	22.19 (1.03)	22.99 (1.38)
OS+OD	20.99 (1.42)	20.67 (1.19)	21.48 (1.68)	22.98 (1.40)	22.20 (1.17)	23.97 (2.15)	24.04 (2.02)	23.06 (1.87)	25.49 (2.92)	24.24 (1.40)	23.38 (1.18)	26.41 (1.93)	24.30 (1.40)	23.67 (1.36)	26.34 (2.26)	24.75 (2.10)	24.11 (1.75)	26.81 (2.25)	24.15 (1.73)	23.21 (1.51)	24.93 (1.83)
	20.20 (0.93)	20.19 (0.85)	20.41 (1.00)	21.78 (0.80)	21.29 (0.78)	21.94 (1.32)	22.45 (1.32)	21.68 (0.92)	22.77 (1.45)	22.88 (1.16)	22.08 (1.03)	23.37 (1.48)	23.48 (1.28)	22.48 (0.98)	23.92 (1.65)	24.19 (1.78)	22.98 (1.58)	24.40 (1.85)	23.80 (1.92)	22.44 (1.59)	23.42 (1.55)
	19.49 (1.00)	19.28 (0.91)	19.60 (0.91)	21.31 (0.77)	20.61 (0.70)	21.13 (0.86)	21.89 (1.18)	20.82 (0.71)	21.60 (1.11)	21.91 (0.90)	20.70 (0.75)	21.74 (1.03)	22.77 (1.45)	21.42 (1.05)	22.24 (1.08)	24.10 (1.92)	22.14 (1.40)	23.30 (1.64)	22.89 (1.59)	21.98 (1.11)	22.76 (1.36)

Supplementary Table 13 Normative longitudinal total retinal thickness (TRT) thickness maps for each block, for the right (OD) and left (OS) eyes separately, as well as thickness values obtained by combining both eyes' data (OD and OS) of WT mice. Data are represented in µm as mean (standard deviation).

	1 Month			2 Months			3 Months			4 Months			8 Months			12 Months			16 Months		
OS	202.75	199.76	198.23	196.34	192.14	191.54	197.36	192.30	191.50	196.58	191.48	191.14	195.46	190.11	188.83	192.94	188.56	187.01	191.71	187.55	184.97
	(3.82)	(4.03)	(3.68)	(4.50)	(3.79)	(3.96)	(4.84)	(4.15)	(3.93)	(3.86)	(3.08)	(3.14)	(4.11)	(4.01)	(4.36)	(4.35)	(3.68)	(3.86)	(6.50)	(6.44)	(4.47)
	213.58	212.84	212.25	205.81	203.64	202.96	205.88	203.51	203.30	205.21	203.06	202.83	204.84	202.31	200.95	204.20	202.01	199.71	204.34	201.72	199.24
	(3.67)	(3.94)	(4.10)	(3.55)	(3.60)	(3.70)	(2.94)	(2.66)	(2.60)	(3.20)	(2.87)	(3.03)	(3.68)	(3.84)	(4.30)	(5.09)	(5.42)	(4.72)	(5.51)	(5.84)	(5.15)
	214.83	214.75	217.65	208.29	207.98	210.26	207.56	206.88	208.98	205.30	204.22	207.56	205.15	204.45	206.33	206.16	205.24	207.33	206.95	205.90	207.65
	(3.92)	(4.86)	(4.19)	(3.31)	(3.39)	(3.52)	(2.79)	(4.28)	(3.81)	(2.85)	(2.45)	(3.43)	(4.10)	(3.80)	(3.71)	(5.14)	(4.94)	(4.23)	(4.75)	(5.25)	(4.57)
OD	203.56	199.76	198.21	195.83	191.46	190.34	194.55	190.19	190.15	195.57	190.20	190.56	195.28	188.45	187.55	193.79	187.94	187.58	190.12	186.34	187.25
	(3.95)	(3.54)	(3.74)	(3.67)	(3.16)	(3.56)	(4.66)	(3.53)	(2.88)	(3.79)	(2.98)	(2.88)	(4.78)	(4.10)	(4.13)	(5.90)	(4.63)	(4.70)	(4.57)	(7.00)	(9.51)
	214.50	213.22	212.34	206.76	204.34	203.13	206.07	203.69	201.86	205.49	202.58	201.67	204.37	201.21	199.44	205.58	201.91	200.23	203.47	201.06	200.32
	(3.71)	(3.83)	(3.83)	(3.13)	(3.47)	(4.27)	(4.22)	(4.21)	(2.29)	(2.98)	(2.91)	(2.98)	(4.02)	(4.27)	(4.31)	(5.39)	(5.97)	(6.67)	(5.69)	(4.89)	(5.43)
	214.90	215.37	218.01	209.55	209.37	210.75	208.37	208.46	209.10	206.50	206.59	208.16	205.22	204.99	207.29	207.58	206.34	209.56	206.69	206.47	209.60
	(3.34)	(3.56)	(3.46)	(3.75)	(3.56)	(3.54)	(3.03)	(3.23)	(3.10)	(3.34)	(3.55)	(3.32)	(3.97)	(4.20)	(4.01)	(5.38)	(5.20)	(6.24)	(5.57)	(5.04)	(4.94)
OS+OD	203.17	199.76	198.22	196.09	191.80	190.95	195.90	191.21	190.82	196.08	190.84	190.85	195.37	189.26	188.18	193.39	188.23	187.32	190.94	186.99	186.21
	(3.89)	(3.78)	(3.69)	(4.10)	(3.49)	(3.80)	(4.93)	(3.97)	(3.49)	(3.84)	(3.08)	(3.01)	(4.44)	(4.12)	(4.27)	(5.22)	(4.21)	(4.31)	(5.66)	(6.67)	(7.64)
	214.05	213.03	212.29	206.29	203.99	203.05	205.97	203.60	202.57	205.35	202.82	202.25	204.60	201.74	200.19	204.90	201.96	199.97	203.87	201.40	199.77
	(3.70)	(3.87)	(3.95)	(3.36)	(3.54)	(3.98)	(3.63)	(3.52)	(2.54)	(3.08)	(2.88)	(3.05)	(3.84)	(4.08)	(4.35)	(5.26)	(5.68)	(5.76)	(5.59)	(5.37)	(5.28)
	214.87	215.06	217.83	208.91	208.68	210.51	207.97	207.67	209.04	205.90	205.40	207.86	205.19	204.72	206.81	206.88	205.78	208.45	206.82	206.21	208.61
	(3.62)	(4.25)	(3.82)	(3.58)	(3.53)	(3.52)	(2.93)	(3.86)	(3.45)	(3.15)	(3.26)	(3.37)	(4.02)	(3.99)	(3.87)	(5.29)	(5.07)	(5.42)	(5.15)	(5.11)	(4.82)
